# YIV-906 potentiated anti-PD1 action against hepatocellular carcinoma by enhancing adaptive and innate immunity in the tumor microenvironment

**DOI:** 10.1038/s41598-021-91623-3

**Published:** 2021-06-29

**Authors:** Xiaochen Yang, Wing Lam, Zaoli Jiang, Fulan Guan, Xue Han, Rong Hu, Wei Cai, William Cheng, Shwu-Huey Liu, Peikwen Cheng, Yuping Cai, Nicholas J. W. Rattray, Caroline H. Johnson, Lieping Chen, Yung-Chi Cheng

**Affiliations:** 1 Department of Pharmacology, Yale University School of Medicine, 333 Cedar Street, SHM B254, New Haven, CT 06510 USA; 2 Department of Cardiology and Health Care, Guanganmen Hospital, China Academy of Chinese Medical Sciences, Beijing, China; 3 Department of Immunobiology, Yale University School of Medicine, New Haven, CT 06510 USA; 4 Yiviva, Inc, 447 Broadway, 2F, New York, NY USA; 5 Environmental Health Sciences, Yale University School of Public Health, New Haven, CT 06510 USA; 6 Strathclyde Institute of Pharmacy and Biomedical Sciences, University of Strathclyde, 161 Cathedral Street, Glasgow, G4 0RE UK

**Keywords:** Cancer, Cancer, Drug discovery

## Abstract

YIV-906 (PHY906) is a standardized botanical cancer drug candidate developed with a systems biology approach—inspired by a traditional Chinese herbal formulation, historically used to treat gastrointestinal symptoms including diarrhea, nausea and vomiting. In combination with chemotherapy and/or radiation therapy, preclinical and clinical results suggest that YIV-906 has the potential to prolong survival and improve quality of life for cancer patients. Here, we demonstrated that YIV-906 plus anti-PD1 could eradicate all Hepa 1–6 tumors in all tumor bearing mice. YIV-906 was found to have multiple mechanisms of action to enhance adaptive and innate immunity. In combination, YIV-906 reduced PD1 or counteracted PD-L1 induction caused by anti-PD1 which led to higher T-cell activation gene expression of the tumor. In addition, YIV-906 could reduce immune tolerance by modulating IDO activity and reducing monocytic MDSC of the tumor. The combination of anti-PD1 and YIV-906 generated acute inflammation in the tumor microenvironment with more M1-like macrophages. YIV-906 could potentiate the action of interferon gamma (IFNg) to increase M1-like macrophage polarization while inhibiting IL4 action to decrease M2 macrophage polarization. Flavonoids from YIV-906 were responsible for modulating IDO activity and potentiating IFNg action in M1-like macrophage polarization. In conclusion, YIV-906 could act as an immunomodulator and enhance the innate and adaptive immune response and potentiate anti-tumor activity for immunotherapies to treat cancer.

## Introduction

Hepatocellular carcinoma (HCC) is the most prevalent and aggressive type of liver cancer^1^. The median prognosis of patients with unresectable and recurrent HCC is only 3 to 7 months^2^. Sorafenib and Lenvatinib, which are multi-kinase inhibitors, were approved by the FDA as the first-line drug for HCC treatment^3^. Sorafenib and Lenvatinib have progression-free survival times of about 3.7 months and 7.4 months with serious adverse effects^4^. In 2017, the FDA granted Nivolumab (anti-PD1) accelerated approval to treat hepatocellular carcinoma (HCC) patients who do not respond to sorafenib. The objective response rate of Nivolumab was 20% and the percentage of patients with a complete response was only 1%, which is a significantly lower rate than for melanoma patients treated with Nivolumab^5^. Additionally, 32% of HCC patients did not even respond to Nivolumab. The therapeutic index for HCC patients treated with Nivolumab can be further improved.

YIV-906 is a standardized four-herb formulation based on an 1800-year old Chinese herbal formulation called “Huang Qin Tang”, which is prescribed to treat numerous gastrointestinal (GI) symptoms, including diarrhea, nausea, and vomiting. The highly refined concoction is composed of four herbs: *Glycyrrhiza uralensis* Fisch (G), *Paeonia lactiflora* Pall (P), *Scutellaria baicalensis* Georgi (S), and *Ziziphus jujuba* Mill (Z). Over a period of 18 years, six batch-to-batch consistent preparations of YIV-906 have been manufactured using cGMP standards and validated using quality control platforms including phytomics^6^ and a recently developed mechanism-based quality control platform (Mech QC) to measure bioequivalence^7^.

In seven Phase I/II to II clinical studies in liver, pancreatic and colorectal cancer at research institutions, including Yale University, Stanford University, UPMC Hillman Cancer Center and City of Hope Comprehensive Cancer Center, results from over 170 patients receiving irinotecan, capecitabine or chemo-radiation^8–11^ in combination with YIV-906, reported no YIV-906-related toxicity at the dosage used along with zero-to-minimal Grade 3/4 non-hematological toxicities, including diarrhea, nausea, vomiting, fatigue—demonstrating improved quality of life as well as survival for patients.

In preclinical studies, YIV-906 was shown to enhance the anti-tumor activity of different classes of anti-cancer agents, including immune checkpoint inhibitors, tyrosine kinase inhibitors, topoisomerase inhibitors, anti-microtubule agents, alkylating agents, antimetabolites, and radiation in vivo^12^. YIV-906’s mechanism of action for enhancing a broad spectrum of anti-cancer agents is primarily due to the induction of acute inflammation within the tumor microenvironment where infiltrated macrophages expressing more M1-like over M2-like phenotypes in the presence of a neo-antigen^13,14^. YIV-906 reduced irinotecan (CPT-11)-induced intestinal inflammation by inhibiting NFκB, COX-2, and iNOS while promoting intestinal stem/progenitor cell repopulation by simulating the Wnt signaling pathway^15^. In irradiation^16^ studies, YIV-906 also reduced GI toxicity. YIV-906 could selectivity alternate the intestines’ bacterial population; however, the microbe change is not responsible for YIV-906’s action on irinotecan^17^.

In this report, YIV-906 shows that it is an immunomodulator that can enhance the anti-tumor activity of anti-PD1 by promoting both adaptive and innate immunity through multiple mechanisms of actions via systems biology effect. Our results suggest YIV-906 may serve as a multitarget immune enhancer in the tumor microenvironment for immunotherapy for the treatment of HCC and other cancers.

## Results

### YIV-906 enhanced anti-PD1 action to inhibit Hepa1-6 tumor growth in vivo and demonstrated tumor-specific vaccine-like effect

**Figure 1:**
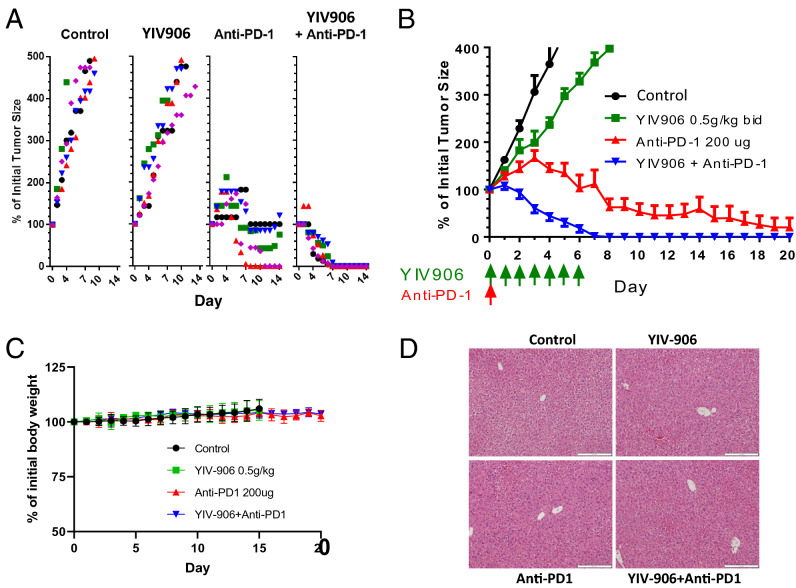
Effect of YIV-906 on the anti-tumor of Anti-PD1 (YIV-906 500 mg/kg p.o. bid × 7, Anti-PD-1 antibody 200ug/mice i.p. once) against Hepa 1–6 tumor growth of C57BL6 mice. (**A**) Individual tumor growth (indicated as spot plot with different colors and symbols) of each treatment group during day 0 to 14. (**B**) Average(± SD) tumor growth of each treatment group during day 0 to 20. Tumor size at the beginning were about 180mm^3^. (**C**) Effect of YIV-906 and/or Anti-PD1 on body weight of animals. (**D**) Hematoxylin and eosin staining for visualizing the formalin fixed sections of liver tissue at the end of the treatments (Scale bar = 50 µm). Details of experimental procedures are given in Materials and Methods.

YIV-906 alone had no effect on Hepa1-6 tumor growth in vivo (P > 0.05) (Fig. 1A,B). Anti-PD1 alone started to slow down tumor growth of Hepa 1–6 on day 4 (Fig. 1A,B). Some tumors were shrunk on day 8 and 40% tumors were below detection limit at the end of the experiment (Fig. 1A,B).

Tumors responded to the YIV-906 plus anti-PD1 in as soon as 2 days with all tumors disappearing following 7-days of treatment (P < 0.001) (Fig. 1A,B). Without further treatment up to 21 days later, no tumors re-appeared in the YIV-906 plus anti-PD1 combination group. YIV 906 plus anti-PD1 did not induce the animals’ body weight loss (Fig. 1C) and did not caused histological changes of normal liver tissues (Fig. 1D) as comparing to the control or single treatment groups. This suggested that YIV-906 plus anti-PD1 did not cause adverse side effect to animals while this treatment can selectively cure Hepa 1–6 tumors in vivo (Fig. 1A,B). When Hepa 1–6 cells were re-implanted into the “cured” mice no tumor growth was found, while naïve mice had tumor growth (data not shown). When CMT167 cells (small cell lung carcinoma) or Pan02 (Pancreatic Ductal Adenocarcinoma) were implanted into the “cured” mice after being re-challenged with Hepa 1–6, CMT167 or Pan02 tumor growth could be observed. This behavior suggested YIV-906 in combination with anti-PD1 could create a tumor-specific vaccine-like effect.

In addition, YIV-906 could potentiate the action of anti-PD1 (70 µg/animal and 200 µg/animal) (Fig S1). It should be noted that the YIV-906 and anti-PD1 (70 µg/animal) combination had a stronger anti-tumor effect than anti-PD1 200 µg/kg (Fig S1). Therefore, YIV-906 could reduce the usage of anti-PD1 by at least threefold while achieving similar or better anti-tumor effects than anti-PD1 alone.

### Combination of YIV-906 and anti-PD1 reduced immune suppression by reducing the PD1 and PDL1 protein expression of Hepa 1–6 tumor

Since tumor shrinking happened on day 4 following YIV-906 plus anti-PD1, we compared apoptosis and DNA damage of tumors at this time point. YIV-906 plus anti-PD1 could significant increased levels of cleaved caspase 9 and DNA breakage (P-H2AX-ser139) (Fig S2A, D, E). The above result supported that YIV-906 enhanced anti-PD1 action to lead to more cell death and tumor shrinkage.

**Figure 2:**
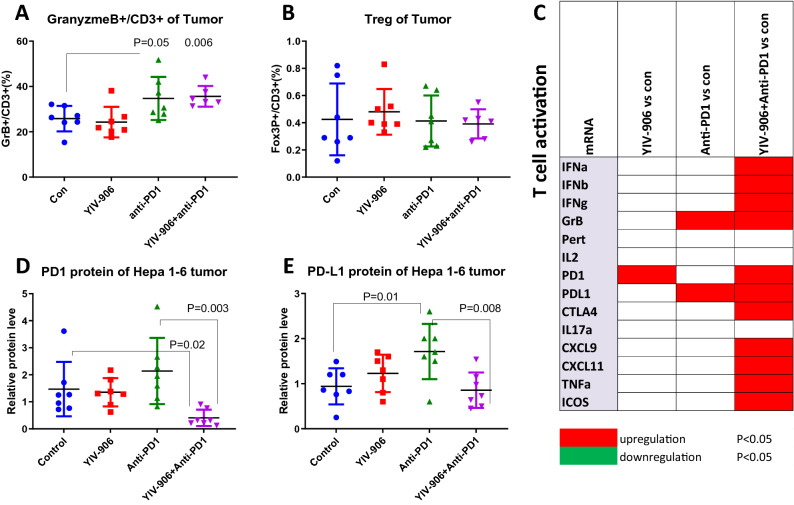
Effect of YIV-906 and/or Anti-PD1 on PD1/PDL1 protein expression and T cell activity. (**A**) Effect of YIV-906 and/or Anti-PD1 on activated T cell of Hepa 1–6 tumor as indicated by Granyzme B and CD3 staining. (**B**) Effect of YIV-906 and/or Anti-PD1 on Treg cell of Hepa 1–6 tumor as indicated by CD3 + /FOX3P + . Following 4 day treatment, tumor tissues were digested by dispase and subsequently stained with fluorescence labelled anti-FOX3P or anti-Granyzme B together with CD3(T cells) and CD45(blood cells). Flow cytometer analysis was used to determined the percentage of Treg or GranyzmeB + ve cells of total T cells. (**C**) Effect of YIV-906 and/or Anti-PD1 on mRNA expression related to T cell of Hepa 1–6 tumor. Using qRT-PCR. T-test P values were shown in the graph. The PD1 (**D**) and PDL1 (**E**) protein expression of Hepa 1–6 tumor following YIV-906 and/or Anti-PD1 treatment. Western blot analysis for the PD1 and PD-L1 protein expression of Hepa 1–6 tumor following 4 days treatment of Anti-PD1-/ + YIV-906. Beta-actin was used for normalization of protein loading. Each sample was normalized to a master mix sample (MIX) which loading duplicated in each gel. Details of experimental procedures are given in Materials and Methods.

The essential function of anti-PD1 is to restore cytotoxic T-cell function by inhibiting the co-inhibitory pathways of T cells through interrupting the interactions between PD1-PDL1^18^. As expected, anti-PD1 induced the number of activated T cells (GranyzmeB + /CD3 +) of Hepa 1–6 tumors (Fig. 2A). The number of activated T cell and Treg upon anti-PD1 treatment was not affected by the co-treatment of YIV-906 (Fig. 2B). Interestingly, the combination treatment did induce more T cell activation related genes in Hepa 1–6 tumors (Fig. 2C), suggesting that the function of T cells could be enhanced. We wondered if YIV-906 had an impact on PD1 and PDL1 protein expression leading to stronger T cell function. Results indicated that Anti-PD1 or YIV-906 alone did not change the PD1 tumor proteins (Fig. 2D). Compared to the control group or anti-PD1 alone group, YIV-906 plus anti-PD1 could significantly decrease PD1 tumor proteins (P = 0.02 or 0.003, respectively) following 4-day treatments (Fig. 2D). This result partially helps explain why less anti-PD1 combined with YIV-906 was required to have similar anti-tumor activity versus taking a higher dosage of anti-PD1 alone. Additionally, anti-PD1, but not YIV-906-only treatment, did significantly increase PDL-1 tumor protein (P = 0.01) but this increase could be counteracted by combining YIV-906 and anti-PD1 (P = 0.008) (Fig. 2E). These results suggested that YIV-906 might facilitate anti-PD1 action in overcoming tumor resistance to immune surveillance and lead to a stronger anti-tumor effect. Furthermore, anti-PD1 plus YIV-906 showed only moderate effects in slowing down the Hepa 1–6 tumor growth in nude mice (T cell deficient) (Fig S3). The synergistic effect between anti-PD1 and YIV-906 did require T cells involvement.

### YIV-906 could modulated IDO activity and decreased myeloid derived suppressor cells (MDSC) of tumor

**Figure 3:**
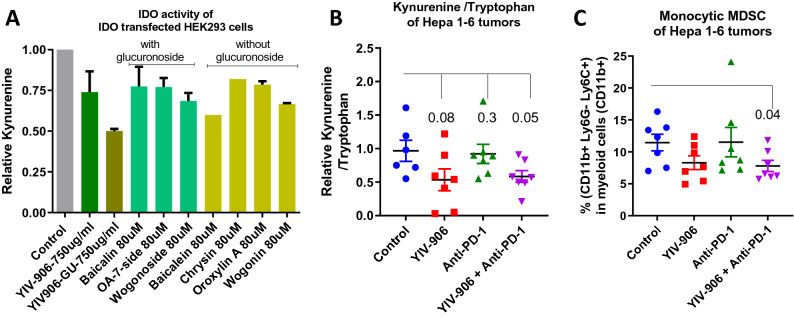
Effect of YIV-906 on IDO activity in vitro and in vivo. (**A**) Effect of YIV-906, E.coli glucuronidase treated YIV906 (YIV906GU), and its flavonoids on IDO activity of IDO transfected HEK-293 cells in culture. HEK-293 cells were transfected with mouse IDO expression plasmids and then seeded for culturing overnight. l-tryptophan 125 µM with or without YIV906, YIV906GU or its flavonoids were added to the wells for 24 h. The concentration of kynurenine of culture medium was measured using colorimetric based assay. Results were normalized to protein concentration in each well. (**B**) Effect of different treatments on Kynurenine/Tryptophan of Hepa 1–6 tumors. (**C**) Effect of different treatment on monocytic MDSC of Hepa 1–6 tumors. P values from T-test are indicated in the **B**,**C**.

IDO (Indoleamine 2,3-dioxygenase) is an enzyme responsible for metabolizing L-tryptophan into kynurenine. IDO expression could regulate T-cells (Tregs)^19^ which help recruiting MDSCs into tumors to cause immune tolerance^20^. IDO could be a key resistance factor to anti-PD1 therapy^20,21^. We found that YIV-906 could modulate IDO activity in cell cultures (Fig. 3A). After using purified *E.coli* glucuronidase (GU) to remove glucuronoside from chemicals to mimic the condition happening in lower GI, YIV-906GU had stronger IDO inhibition than YIV-906 (Fig. 3A). Baicalein was shown to be the most potent compound among the flavonoids (Fig. 3A). It was found that YIV-906 or YIV-906/anti-PD1 had lower kynurenine/tryptophan ratio of Hepa 1–6 tumor (Fig. 3B). This suggested that YIV-906 could modulate IDO activity in vivo. Furthermore, we found that anti-PD1 plus YIV-906 treatment reduced monocytic MDSC of Hepa 1–6 tumor (Fig. 3C). Modulation of IDO by YIV-906 could be an additional mechanism action to reduce immune tolerance and facilitate the action of Anti-PD1.

### YIV-906 plus anti-PD1 treatment induced macrophage infiltration and polarized to a higher M1-like macrophage signature in Hepa 1–6 tumors

**Figure 4:**
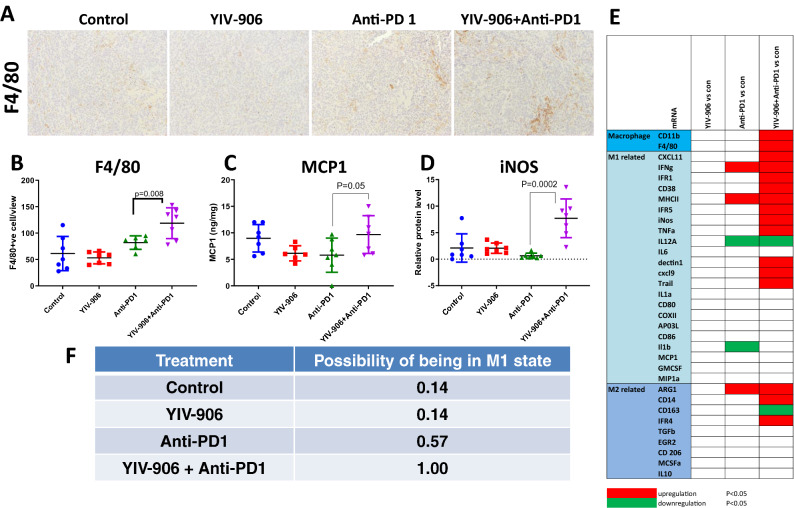
Impact of YIV906 and/or Anti-PD1 on macrophages and M1/M2 signature genes expression of Hepa 1–6 tumor. (**A**) Immunohistochemistry staining of F4/80 for macrophage infiltration into Hepa 1–6 tumor after 4-day treatments. (**B**) Quantification of macrophage of tumor sections after 4-day treatments. (**C**,**D**) MCP1 and iNOS protein expression of Hepa 1–6 tumor after 4-day treatments. (**E**) Heat map (significantly be up-regulated: red, significantly by down regulated: green) for indicating the mRNA expression determined by RT-qPCR following treatment at day 4. (**F**) Possibility of being M1 state based on the signature gene expression of (**E)**. P values were obtained from T-test analysis. Details of experimental procedures are given in “Materials and methods”.

The combination of YIV-906 plus anti-PD1, but not YIV906 alone or anti-PD1 alone, could significantly induce macrophage infiltration in Hepa 1–6 tumors after 4-days of treatment (Fig. 4A,B). This could be attributed to the increase of MCP1(CCL2), a monocyte chemoattractant protein, of tumors in the YIV-906 plus Anti-PD1 treatment group where MCP1 was higher than that of the anti-PD1 only group (P < 0.05) (Fig. 4C and S4A). Depending on the tissue microenvironment, macrophages can be differentiated into two distinct phenotypes: M1 (tumor rejection) and M2 (tumor promotion). Based on bio-statistical analysis, YIV-906 plus anti-PD1 treatment had tumor environment favoring M1-like macrophages (Fig. 4E,F). Western blot analysis further confirmed that the iNOS protein (a M1 marker) but not ARG protein (a M2 marker) was substantially increased following YIV-906 plus anti-PD1 treatment (Fig. 4D and S5A–C). This result also suggested that YIV-906 plus anti-PD1 treated tumors were highly inflamed. It is known that IFNg helps polarize macrophages into the M1-like state. We found that INFg mRNA expression of tumors was significantly induced by YIV-906 plus anti-PD1 treatment (Fig. 4E and S6C). Therefore, the enhanced infiltration of M1-like macrophages induced by YIV-906 plus Anti-PD1, could be an additional mechanism aiding against Hepa1-6 tumor growth.

### YIV-906 could potentiate IFNg to polarize bone marrow derived macrophages (BMDM) into M1-like macrophage while inhibiting IL4 to polarize BMDM into M2-like macrophage in vitro

**Figure 5:**
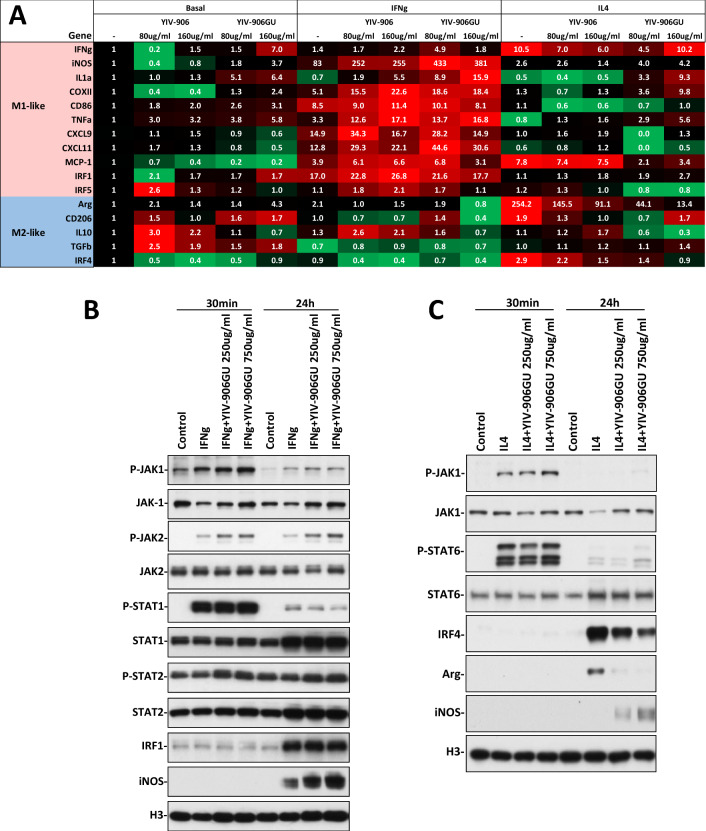
Effect of YIV-906 on the action of INFg or IL4 on polarizing bone marrow derived macrophage (BMDMs) into M1 or M2-like macrophage. (**A**) Heat map for the mRNA expression levels of BMDM following INFg or IL14 with or without YIV-906/GU treatment. For each row (gene), up-regulation of mRNA is highlighted as (red) while down-regulation is highlighted as (green). Number of the table shows the relative fold change gene expression in each treatment conditions (average of three independent experiment and all gene expressions were normalized to actin). (**B**) Western blot analysis for the effect of YIV-906GU on the action of IFNg on IFNg signaling of BMDMs. Cropped blots are used in this figure and they have been run under the same experimental conditions. (**C**) Western blot analysis for the effect of YIV-906GU on the action of IL4 on IL4 signaling of BMDMs. Bone marrow cells were cultured in the presence of murine M-CSF (10 ng/ml) for 7 days, and then cultured in presence with IFNg 10 ng/ml to induce polarization to M1-like macrophage while M2 like macrophage were induced by IL-4 20 ng/ml for 24 h. YIV-906 or YIV-906GU was added at the same time with IFNg or IL4. The mRNA expression of M1 or M2 related genes were determined by RT-qPCR. Protein expression or phosphorylation were detected with western blotting. Histone H3 was used for normalization of protein loading. Cropped blots are used in this figure and they have been run under the same experimental conditions. Details of experimental procedures are given in “Materials and methods”.

As YIV-906 could promote more M1-like macrophage polarization, we investigated if YIV-906 had direct impact on polarizing BMDM into either M1-like or M2-like phenotype in culture. We first examined if β-glucuronidase treatment (GU), which could catalyze hydrolysis of β-d-glucuronic acid residues from certain chemicals could affect the macrophage polarization activity of YIV-906. The results indicated that YIV-906GU had a stronger induction effect on IFNg, IL1a, TNFa mRNA expression of BMDM than YIV-906 alone (Fig. 5A). Furthermore, YIV-906 could potentiate IFNg to polarize BMDM into M1 macrophages with increased expression signals of iNOS, MCP-1, CXCL9, CXCL11, COXII, IL1a, TNFa and CD86 (Fig. 5A). GU treatment further enhanced the potentiation activity of YIV-906 on iNOS, IL1a, CXCL11 (Fig. 5A).

Conversely, YIV-906 could inhibit the action of IL4 for M2 macrophage polarization exhibited by the decreasing mRNA expression levels of Arginase (Arg), CD206 and IRF4 (Fig. 5A). GU treatment could further increase the inhibitory activity of YIV-906 on Arg, IL10 and IRF4 mRNA expression in the presence of IL4 (Fig. 5A).

We further studied if YIV-906 could affect the downstream events of the signaling cascade triggered by IFNg or IL4. In the presence of IFNg, YIV-906GU could further enhance P-Jak1/2 and P-Stat2 protein in as early as 30 min. It could maintain higher P-Stat2 at 24 h in the presence of IFNg in BMDM. At 24 h YIV-906 or YIV-906GU potentiated IFNg in inducing iNOS protein expression but not the IFR1 protein of BMDM (Fig. 5B, S7 and S8). This could be because IFR1 might already have reached its maximum level at the given concentration of IFNg. In addition, IL15RA and ICAM mRNA could also be up-regulated by YIV-906GU in the presence of IFNg in BMDM (Fig S9A and S9B). YIV-906 or YIV-906GU potentiated IFNg action not limited to BMDM. but could also potentiate IFNg to induce MCP1, TNFa, iNOS mRNA in GM-CSF treated Raw cell 264.7 (macrophages) (Fig S10).

In contrast to IFNg, YIV-906 or YIV-906GU inhibited IL4 action by suppressing IRF4 expression, a key transcription factor of the IL4 signaling pathway (Fig. 5C, S11 and S12). This also led to down-regulation of Arg protein in BMDM (Fig. 5C, S11 and S12). The decrease of IFR4 and Arg protein could be attributed to down-regulation of their mRNA by YIV-906 or YIV-906GU in the presence of IL4 (Fig. 5A).

Our results demonstrated that YIV-906 or YIV-906GU could also potentiate IFNg action by stimulating P-Jak1/2 and P-Stat2 phosphorylation while inhibiting IL4 action by down-regulating IFR4 protein of BMDM. The modality could explain how multiple mechanisms of YIV-906 can work to polarize macrophages into the M1-like phenotype. The immuno-modulatory effect of the above activities could be explained by the sugar moiety of chemicals present in YIV-906, specifically the aglycone chemicals which appear most active.

### Flavonoids play key roles of YIV-906 in potentiating IFNg action to polarize macrophages into M1-like type

**Figure 6:**
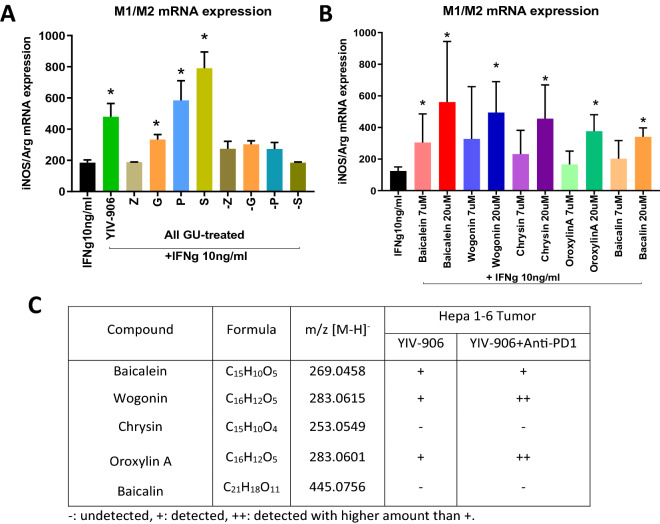
Effect of flavonoids of YIV-906 in potentiating IFNg action. (**A**) Effect of YIV906GU, single herbs (G, P, S and Z: GU treated) or one herb deleted formulation (-G, -P, -S and -Z: GU treated) on the mRNA expression of iNOS/Arg of macrophage. (**B**) Effect of baicalein, wogonin, chrysin, oroxylin A and baicalin on the mRNA expression of iNOS/Arg of macrophage. Raw cells were cultured in the presence of murine M-CSF (10 ng/ml) for 3 days, and then cultured in presence with IFNg 10 ng/ml alone or with YIV-906GU/its components to induce polarization to M1-like macrophage for 24 h. The mRNA expression was determined by RT-qPCR. P-value (T-test) less than 0.05 (Treatment vs control) was highlighted with (*) in the figure. (**C**) Detection of YIV-906 compounds of Hepa 1–6 tumor following oral administration of YIV-906 with or without Anti-PD1 using LC–MS. Details of experimental procedures are given in “Materials and methods”.

The key YIV-906GU components responsible for potentiating the IFNg action on macrophages were investigated. Of the four herb ingredients in YIV-906GU: G, P, S and Z, results indicated that G, P, S, in the presence of IFNg, could increase iNOS/Arg mRNA expression ratio (S > P > G) (Fig. 6A). Consistently the formulations without G, P or S (-G, -P, -S) completely lost the IFNg potentiation property (Fig. 6A). These results indicated that G, P, Z could also play a role in the IFNg potentiation or interact with S to enhance IFNg action. In addition, key flavonoids (baicalein wogonin, chrysin, oroxylin A and baicalin) of S^22,23^ could increase the IFNg action to increase iNOS/Arg mRNA expression ratio (Fig. 6B).

Most importantly, the amount of wogonin and oroxylin A in the tumors was higher in the YIV-906 plus anti-PD1 group compared to the YIV-906 alone group (Fig. 6C, Table S2). Thus, these flavonoid compounds naturally present in component S of YIV-906 could be the active ingredients, along with others, contributing to the IFNg potentiation that polarizes macrophages to the M1-like phenotype in Hepa 1–6 tumors.

## Discussion

Many current immune therapies for cancer are trying to convert "cold tumors" into "hot tumors" so that revived immune cells could attack tumor cells. Immune check point antibodies, such as anti-PD1, anti-PD-L1, anti-CTLA4 have led to breakthroughs for the treatment of many tumor types; However, tumor types such as HCC, pancreatic and colon cancers have had relatively low response rates to these antibodies. Currently hundreds of remedies are being tested to see if they can further improve the efficacy of these immune check point antibodies. Many of these new remedies are designed to target a specific target (vs multiple targets) of the immune cycle. Up to date, there have been no major breakthroughs in these combination clinical trials. Here, we reported that YIV-906, a botanical immunomodulator with a systems biology effect, could potentiate anti-PD1 action against Hepa 1–6 tumor growth by promoting both adaptive and innate immunity through multiple mechanisms of actions.

For adaptive immunity, we found YIV-906 plus anti-PD1 could decrease PD1 tumor proteins and inhibited PDL-1 expression induced by anti-PD1. This could foster a more favorable tumor microenvironment for T cell activation. In addition, we found that YIV-906 could modulate IDO activity and leading to a decrease of MDSC of Hepa 1–6 tumor. We identified that flavonoids of S herb played a key role in modulating IDO activity. This result was supported by a previous report^24^. IDO inhibitors were reported to enhance the action of anti-PD1, anti-PD-L1, anti-CTLA4 on different types of animal tumors^20,21,25^. BMS-986205, with less side effects compared to Epacadostat which had serious adverse effects^26^ and failed in clinical trials^27^, is still being tested in combination with Nivolumab as first or second line therapy for liver cancer (NCT03695250).

In addition to adaptive immunity, YIV-906 also enhances the innate immune response. YIV-906 plus anti-PD1 could attract more M1-like macrophage infiltration which could be partly due to the induction of MCP1 in the tumors. Interestingly, YIV-906 also increased M1-like macrophage tumor infiltration when combined with irinotecan (CPT-11) or Sorafenib^13,14^. There is increasing evidence to support M1-like macrophages in tumors could enhance the efficacy of chemotherapy and target therapies^13,28,29^. It has also been reported that M1-like macrophages phenotype help T cells re-activation under immune checkpoint blockade therapy^30^. PD1 expression was found to dictate macrophage polarization. M2 macrophages, which have high PD1 expression and low phagocytic activity, promote tumor growth and are not favorable for immunotherapy^31^. In contrast, low PD1 expression favors M1-like macrophages that have high phagocytic activity and could increase immune check point blockade therapy action^31,32^. A recent report demonstrated that anti-PD1 could help switch macrophage polarity states from M2 to M1-like phenotypes in lung cancer^33^. Our result also indicated that anti-PD1 alone increased the probability of M1-like macrophage in the tumor microenvironment by 40%. Most importantly YIV-906 combined with anti-PD1 could further enhance M1-like macrophages and the innate immune response in the tumor microenvironment. YIV-906 plus anti-PD1 even further decreased PD1 proteins in tumor tissues which subsequently created favorable conditions for M1-like macrophage polarization^31^. The decrease in PD1 protein levels in the YIV-906 plus anti-PD1 group also explains why lower dosages (by 1/3), of anti-PD1 combined with YIV-could also achieve the same anti-tumor activity for higher doses of anti-PD1.

The combination not only eradicated the Hepa 1–6 tumors in every mouse, it also mimicked tumor-specific vaccine-like behavior as demonstrated by selective rejection of re-implanted Hepa 1–6 tumors and the growth of implanted CMT167 or Pan02 tumors. Some studies suggest that boosting innate immunity could increase tumor vaccine effect. A recent report demonstrated that exosomes secreted by M1-like macrophages can potentiate cancer vaccine-like properties through Th1 cytokine induction^34^ resulting in a proinflammatory tumor climate. A separate study reporting that M1-like macrophages function by aiding CD8 T cell differentiation into memory T cells^35^. Our next step would be to investigate the extent exosomes or T-cells play in the vaccine mimicking behavior.

IFNg plays an important role in macrophage M1-like polarization. YIV-906 could potentiate the IFNg activity to turn up the signaling transduction response to a higher level; as anti-PD1 alone could activate T cells which released IFNg in tumor, adding YIV-906 could further amplify the IFNg signal and enhance M1-like macrophage polarization. Another unique property of YIV-906 was the inhibitory activity demonstrated on the M2 inducer, IL4, through down-regulation of IFR4. When treated with the combination of YIV-906 and anti-PD1, the dual effect of promoting M1-like polarity while inhibiting the M2 state ensures the dominance of M1-like macrophages in tumor tissues. Currently, there was no other therapeutic agents that can have these synergistic systems biology properties.

We identified *Scutellaria baicalensis Georgi* (S) as the key herb ingredient that is most likely responsible for promoting M1-like macrophage polarization. This result was consistent with our previous study of the effects of YIV-906 in combination therapy with anti-cancer drugs CPT-11 or sorafenib. Similar elevated predominantly M1-like macrophage signals were observed, as well as the enhanced anti-tumor activity of the combination^13,36^. Flavonoids were identified as the active compounds in S that promotes M1-like macrophage polarization. The flavonoid baicalin’s ability to induce repolarization of tumor-associated macrophages to M1-like phenotype was demonstrated by others^37^. Most importantly, we detected baicalein, wogonin and oroxylin A in the Hepa 1–6 tumor and they could potentiate IFNg in the tumor to polarize macrophages into M1-like.

In conclusion, YIV-906 enhanced the anti-tumor activity of anti-PD1. The action is due to its ability to promote both adaptive and innate immunity. Flavonoids from *Scutellaria baicalensis Georgi* (S) were identified as one of the major active compounds responsible for modulating IDO activity, which regulated MDSC function and potentiated IFNg action to polarize macrophages into M1-like type. It could be interesting to investigate other herbs or herbal formulations containing S or flavonoids on anti-PD1 actions. YIV-906 component herbs G, P, Z might also contribute to the activity of YIV-906 and this is under current investigation. The potential use of YIV-906 as an immunomodulatory enhancer of tumor microenvironment in combination with Anti-PD1, or other immune check point antibodies, for the treatment of HCC or other type of cancers should be explored further in the clinic. Currently, a phase II international clinical trial for using YIV-906 (600 mg (3 capsules) BID, 4 days on 3 days off) plus sorafenib (400 mg BID, daily) had be initialized to target HBV positive hepatocellular carcinoma (NCT04000737). As similar scheduling and dosing (up to 2400 mg, BID) of YIV-906 had been used for several clinical trials with no detection of YIV-906 associated toxicity of patients^8–11^, similar scheduling and dosing of YIV-906 could be combined with anti-PD1 treatments in the future clinical trials.

## Materials and methods

### Animal studies

Details of animal studies can be found in our previous reports^13,15^. Briefly, Hepa 1–6 cells (about 2 × 10^6^ cells in 100 μl phosphate-buffered saline) were transplanted subcutaneously into 8-week-old female C57BL6 mice (Charles River Laboratories, Wilmington, MA). Body weight, tumor size, and mortality of the mice were monitored daily. After 10–14 days, mice with tumor sizes of 180 mm^3^ were selected. Tumor volume was examined by using the formula length × width^2^ × π/6. Each group consisted of seven mice. YIV-906 was administered orally for 7 days (500 mg/kg po, twice per day), while anti-PD1 was administered intraperitoneal on day zero (200ug/mice). In the control groups, mice were administered water orally. On Day 0, YIV-906 was administered 30 min prior to anti-PD1 administration. All animal experiments were carried out in accordance with the relevant guidelines and regulations approved Yale University Institutional Animal Care and Use Committee (IACUC) protocol. Animal experimental protocols were approved by Yale University Institutional Animal Care and Use Committee (IACUC). Animal studies were carried out in compliance with the ARRIVE guidelines.

### Immunohistochemistry

After 4-day treatments, mice were terminated by cervical dislocation two days or four days after initiation of drug treatment (see above). Details of immunohistochemistry protocol can be found in our previous reports^13,15^. Intestinal and colon tissues were removed, fixed in formalin, embedded in paraffin, and sectioned into 10 μm. The sections were mounted on Superfrost slides, dewaxed with xylene, and gradually hydrated. Antigen retrieval was achieved by 10 mM Sodium citrate pH6.0 with 0.02% Tween-20 under steaming for 30 min. The source and dilution of primary antibody are listed in supplementary methods. The primary antibodies were diluted using Tris–HCl buffer containing 1% BSA and 0.5% Tween-20 and were incubated at room temperature for one hour. As a negative control, a set of slides was processed without primary antibody. Super-picture immunohistochemistry detection kit (Invitrogen, Inc.) was used for detection. The slides were counterstained with hematoxylin and mounted. The antibodies used were: Cleaved Caspase-3 (#9664, Cell Signaling Technology, Inc.), Cleaved Caspase-8 (#9496, Cell Signaling Technology, Inc. Danvers, MA), Cleaved Caspase-9 (#ab52298, Abcam, Cambridge, England), F4/80 (#ab16911, Abcam).

### Flow cytometry analysis

Tumor tissues (200 mg) were cut into small pieces in 0.5 ml RPM1 640 culture medium. Liberase was added to dissociate the connected tumor cells at room temperature for 15 min. Dissociated cells were passed through a cell strainer (70um). After spinning down the cells at 1000 g centrifugation for 10 min, red blood cells were lysed with 1 ml BD pharm lyse on ice. Cells were collected at 1000 *g* centrifugation for 10 min. 2 × 10^6^ cells were used for each staining sample. Cells were resuspended in RPM1 640 with 3% FBS. Anti-mouse CD16/CD32 clone 2.4G2 (BD Pharmingen, #553142) was used to block Fc receptors on cells. Total T cells were stained by Anti-CD3-PE (BD Pharmingen, clone 145-2c11, #553064) for 30 min on ice. Fixation/Permeabilized (eBioscience) was used to fix and permeabilize cells. Then activated cytotoxic T cells was further stained with Anti-Granzyme B-pacific blue (BioLegend, clone GB11, #515408) and T regulatory cells were stained with Anti-FOX3P-APC (eBioscience, clone FJK16s, #17-5773-83). The stained cells were washed and analyzed by flow cytometry LSR II (BD Canto II, New Jersey, USA). Details of flow cytometry analysis can be found in our previous report^38^.

### Western blot

BMDM or RAW 264.7 cells (American Type Culture Collection) were cultured RPMI supplemented with 5% FBS in 37 °C incubator with 5% CO_2_. 2 × 10^6^ cells were seeded in 12-well plate. After drug treatment, cells were lysed in 0.3 ml protein loading buffer (for 20 ml buffer, 10% SDS 4 ml, Tris–HCL pH 6.8 0.75 ml, 10% glycerol 5 ml, β-mercaptoethanol 0.5 ml, and bromophenol blue) for each well, and sonicated for 30 s to break DNA. Then cell extracts were electrophoresed through Mini PROTEAN TGX Precast gels (12%, 15 well comb, 15 µl/well Cat. #456-1046) in a running buffer (10 × , Tris 30 g, Glycine 144 g, SDS 10 g, with DD H_2_O) and transferred to the nitrocellulose membrane (Bio-Rad Laboratories, Inc) in a transfer buffer (Tris 30 g, Glysine 144 g, SDS 0.5 g). After blot transfer, membrane was cut into two parts with approximate size of 3 cm (height) x 9 cm (wide) to fit into a blocking chamber. The upper part of the membrane will be used for probing target proteins with specific antibodies (as following), the lower part of the membrane will be used for probing actin or Histone 2 (H3) as protein loading control for normalization. The membrane was blocked and probed in TBS-T buffer (TBST + 1% Tween, AB14330-01000, American Bioanalytical) containing non-fat milk 1:5000 (Blotting-Grade Blocker, Cat. #170-0604 Nonfat dry milk). Primary antibodies (PD-1 (D7D5W) XP Rabbit mAb #84651S Mouse Specific lot:1 Ref: 08/2017) at 1:1000 in TBS-T buffer (TBST + 1% Tween, AB14330-01000, American Bioanalytical) were incubated with the membrane for shaking overnight at 4 °C. Histone, H3 was used as an internal control for normalization and detected with a monoclonal actin antibody diluted at 1:1000 (H3(D1H2) Rabbit mAb #4499S Ref: 06/2017). After washing with TBS-T three times, each time for 5 min, the membranes were then further incubated with goat anti-rabbit IgG-HRP SC-2004, lot #B1711 HRP conjugated 1:5000, and incubated in room temperature for 1 h. Then the membrane was washed with TBS-T three times again. Stable Peroxide Solution 1 ml (SuperSignal West Pico PLUS, Prod#1863097) and Luminol/Enhancer Solution 1 ml (SuperSignal West Pico PLUS, Prod#1863096) were used for visualizing, and scan with densitometer. Antibody list: PD-1 (D7D5W) XP Rabbit mAb #84651S Mouse Specific lot:1 Ref: 08/2017 (Cell Signaling), Anti-PD-L1 antibody [EPR20529]ab213480, Arginase-1 (D4E3M) XP Rabbit mAb#93668 (Cell Signaling), iNOS Antibody (Mouse Specific) #2982 (Cell Signaling), Jak1(6G4)Rabbit mAb#3344 (Cell Signaling), P-Jak1 (Y1034/1035)(D7N4Z) Rabbit mAb#74129 (Cell Signaling), Jak2(D2E12) XP Rabbit mAb #3230(Cell Signaling), P-Jak2(Y1008)(D4A8) Rabbit mAb #8082 (Cell Signaling), Stat1 Antibody#9172(Cell Signaling), Phospho-Stat1(Tyr701)(D4A7)Rabbit mAb#7649(Cell Signaling), Stat2(D9J7L)Rabbit mAb#72604(Cell Signaling), Phospho-Stat2(Tyr690)-R sc-21689 #K1609(SantaCruz), Stat6(D3H4)Rabbit mAb#5397(Cell Signaling), Phospho-Stat6(Tyr641)(D8S9Y)Rabbit mAb#56554(Cell Signaling), IRF-1(D5E4) XP Rabbit mAb #8478(Cell Signaling), IRF-4(D9P5H)Rabbit mAb#15106(Cell Signaling).

### Quantitative real time RT-PCR

Total RNA was extracted with TRIzol reagent (Invitrogen, California, USA). The aqueous phase was collected and then one volume of ethanol was added, following the manufacturer’s instructions. Before centrifuging, this slurry was added to a column (miRNeasy, Qiagen, Venlo, Limburg) for further extraction and simultaneous DNA digestion (RNase-Free DNAse set, Qiagen). cDNA was synthesized using random primers and reverse transcriptase MMLV (New England Biolabs, Ipswich, MA). qPCR assays were performed using iTaq SYBR Green Supermix and the CFX96 Real-Time PCR Detection System (Bio-Rad Laboratories, Hercules, CA). Primer sets were listed in the supplementary method. The mouse primer pair sequences are attached in supplementary methods and β-actin serves as internal control (please see supplementary methods for primer sequences). Relative expression of target genes against β-actin was expressed as 2^−ΔCt^ and fold differences was calculated as expressed mRNA of YIV-906 and or antiPD1-treated samples against untreated samples. Primers sequences are listed (Table S1) and the rest of primers that could be found in our previous reports^13,15^.

### Cytokine analysis by cytometric bead array

Animal plasma and tumor tissue of YIV-906 and or antiPD-1-treated mice and control mice were collected after 96 h following the treatments. Culture medium of untreated and YIV-906-treated BMDMs were collected after 24 h exposure. Determination of cytokine expression (IL-6, MIP-1a, IL-5, IL-17A, IL-12p70, TNFa, IL-1B, IL-10, MIG, IFNg, MCP-1, G-CSF) was performed using cytometric bead array flex set kit by flow cytometry (BD Canto II, New Jersey, USA) according to the manufacturer’s instructions (BD biosciences, UK)^15^.

### Isolation of bone marrow derived monocytes (BMDMs) and macrophage differentiation

Bone marrow cells were collected from tibias and femurs of 10-week-old C57Bl/6 mice were cultured with complete RPMI-1640 medium (supplemented with 5% Fetal Bovine Serum and 1% Penn/Strep) in the presence of murine M-CSF (10 ng/ml) for 7 days to allow differentiation of monocytes into macrophages^39^. Macrophage were cultured in 5% FBS RPMI-1640 medium with IFNg (10 ng/ml) to induce polarization to M1-like macrophage while M2-like macrophage were induced by IL4 (20 ng/ml).

### IDO activity assay

2 × 10^6^ HEK293 cells were transfected with mouse IDO (2 µg/10 cm plate) (OriGene Technologies, Inc., Rockville, MD, Ido1 (NM_008324)) or without IDO DNA as negative control using lipofectamine 3000 for 48 h. For one plate, 1 ml PBS was used to collect cells into a 2 ml tube. Cells were spin down at 3500 rpm 1 min. Cells were then sonicated in ice cold 1 ml PB buffer pH 6.5. Cell lysis was clarified by centrifuging at 12000 rpm for 5 min at 4 °C. 25 µl cell lysis will be mixed with 25 µl herbal extract at desired concentration and 50 µl reaction buffer: (PB buffer 100 mM pH 6.5) every 10 ml add 70 mg Vitamin C, 10 µl methylene blue (2.5%), 100 µl catalase (20 mg/ml), 250 µl of 500 mM l-tryptophan. Mixture was incubated for 1.5 h at 37 °C in water bath. Trichloroacetic acid 30% 25 µl will be added and incubated at 50 °C for 1 h. Finally, Enrlich 0.8% (80 mg/10 ml in acetic acid) 100 µl was added. Absorbance at 540 nm will be measure^40^. Optical density at 540 nm (Yellow) has positive correlation to the amount of kynurenine^40^.

### LC–MS detection

Each tumor sample were homogenized in 200 μL acetronitrile/methanol/water(2/2/1, v/v/v) and 1 mm glass beads (BioSpec Products, Bartlesville, OK) for 30 s at 3500 rpm twice. The homogenate was then centrifuged at 12,000 rpm for 15 min at 4 °C. The supernatant was dried down in a Speedvac. The residue of each tumor sample was re-dissolved in 100 µL of acetonitrile, and vortexed at 3000 rpm for 3 min. The solution was then centrifuged at 12,000 rpm at 4 ℃ for 15 min, and 2 μL supernatant was injected into the UPLC-QTOF system for analysis. All sample analyses were performed on an ACQUITY ultra-performance liquid chromatography (UPLC) system coupled with a quadrupole-time of flight (Q-TOF) MS instrument (UPLC Xevo G2-XS QTOF MS, Waters Corp., Milford, MA, USA) with an electrospray ionization (ESI) source. Separation was carried out on a Waters ACQUITY BEH C18 column (2.1 X100 mm id, 1.7 μ m) with a guard column (Waters ACQUITY BEH C18 column (2.1 X5 mm id, 1.7 μ m)). The mobile phase consisted of acetonitrile (A) and water containing 0.1% formic acid (B) using a gradient elution of 5% A at 0–2 min, 5–10% A at 2–3 min, 10–17% A at 3–10 min, 17–30% A at 10–15 min, 30–40% A at 15–20 min, 40–80% A at 20–25 min, 80% A at 25–30 min, 80–5% A at 30–31 min, and 5% A at 31–35 min. The flow rate was 0.3 mL/min. Mass spectrometry was performed on a Water Xevo G2-XS QTOF. The scan range was from 50 to 1000 Da. For negative electrospray mode, the capillary voltage and cone voltage were set at 2.5 kV and 60 V, respectively. The desolation gas was set to 800 L/h at a temperature of 500 C; The cone gas was set to 50 L/h at a temperature of 120 C; Data acquisition was achieved using MS^E^, and the collision energy was 15–60 V.

### Statistical analysis

Data were analyzed by one- or two-way analysis of variance (ANOVA) (GraphPad Prism 7), correlation analysis (GraphPad Prism 7) and Student’s t test (Microsoft Office Excel). The difference was statistically significant when P < 0.05.

## Supplementary Information

Supplementary Information.

## References

[1] El-Serag HB (2011). Hepatocellular carcinoma. N. Engl. J. Med..

[2] Llovet JM, Bruix J (2008). Molecular targeted therapies in hepatocellular carcinoma. Hepatology.

[3] Wilhelm SM (2004). BAY 43-9006 exhibits broad spectrum oral antitumor activity and targets the RAF/MEK/ERK pathway and receptor tyrosine kinases involved in tumor progression and angiogenesis. Cancer Res..

[4] Kudo M (2018). Lenvatinib versus sorafenib in first-line treatment of patients with unresectable hepatocellular carcinoma: a randomised phase 3 non-inferiority trial. Lancet.

[5] El-Khoueiry AB (2017). Nivolumab in patients with advanced hepatocellular carcinoma (CheckMate 040): An open-label, non-comparative, phase 1/2 dose escalation and expansion trial. Lancet.

[6] Tilton R (2010). A comprehensive platform for quality control of botanical drugs (PhytomicsQC): A case study of Huangqin Tang (HQT) and PHY906. Chin. Med..

[7] Lam W (2018). Mechanism based quality control (MBQC) of herbal products: A case study YIV-906 (PHY906). Front. Pharmacol..

[8] Farrell MP, Kummar S (2003). Phase I/IIA randomized study of PHY906, a novel herbal agent, as a modulator of chemotherapy in patients with advanced colorectal cancer. Clin. Colorectal Cancer.

[9] Saif MW (2010). Phase I study of the botanical formulation PHY906 with capecitabine in advanced pancreatic and other gastrointestinal malignancies. Phytomedicine.

[10] Kummar S (2011). A phase I study of the Chinese herbal medicine PHY906 as a modulator of irinotecan-based chemotherapy in patients with advanced colorectal cancer. Clin. Colorectal Cancer.

[11] Saif MW (2014). First-in-human phase II trial of the botanical formulation PHY906 with capecitabine as second-line therapy in patients with advanced pancreatic cancer. Cancer Chemother. Pharmacol..

[12] Liu, S.H. & Cheng, Y.C. Old formula, new Rx: the journey of PHY906 as cancer adjuvant therapy. *J Ethnopharmacol* **140**, 614–623 (2012) 10.1016/j.jep.2012.01.047 22326673

[13] Lam W (2015). PHY906(KD018), an adjuvant based on a 1800-year-old Chinese medicine, enhanced the anti-tumor activity of Sorafenib by changing the tumor microenvironment. Sci. Rep..

[14] Wang E (2011). Interaction of a traditional Chinese medicine (PHY906) and CPT-11 on the inflammatory process in the tumor microenvironment. BMC Med. Genomics.

[15] Lam, W. *et al.* The four-herb Chinese medicine PHY906 reduces chemotherapy-induced gastrointestinal toxicity. *Sci. Transl. Med.* **2**, 45–59 (2010). 10.1126/scitranslmed.3001270 20720216

[16] Rockwell S (2013). Preclinical studies of the Chinese herbal medicine formulation PHY906 (KD018) as a potential adjunct to radiation therapy. Int. J. Radiat. Biol..

[17] Lam W (2014). The number of intestinal bacteria is not critical for the enhancement of antitumor activity and reduction of intestinal toxicity of irinotecan by the Chinese herbal medicine PHY906 (KD018). BMC Complement. Altern. Med..

[18] Intlekofer AM, Thompson CB (2013). At the bench: Preclinical rationale for CTLA-4 and PD-1 blockade as cancer immunotherapy. J. Leukoc. Biol..

[19] Munn DH, Mellor AL (2007). Indoleamine 2,3-dioxygenase and tumor-induced tolerance. J. Clin. Invest..

[20] Holmgaard RB (2015). Tumor-expressed IDO recruits and activates MDSCs in a Treg-dependent manner. Cell Rep..

[21] Holmgaard RB, Zamarin D, Munn DH, Wolchok JD, Allison JP (2013). Indoleamine 2,3-dioxygenase is a critical resistance mechanism in antitumor T cell immunotherapy targeting CTLA-4. J. Exp. Med..

[22] Zhang W (2010). Identification of chemicals and their metabolites from PHY906, a Chinese medicine formulation, in the plasma of a patient treated with irinotecan and PHY906 using liquid chromatography/tandem mass spectrometry (LC/MS/MS). J. Chromatogr. A.

[23] Ye M (2007). Liquid chromatography/mass spectrometry analysis of PHY906, a Chinese medicine formulation for cancer therapy. Rapid Commun. Mass Spectrom..

[24] Chen S, Corteling R, Stevanato L, Sinden J (2012). Natural inhibitors of indoleamine 3,5-dioxygenase induced by interferon-gamma in human neural stem cells. Biochem. Biophys. Res. Commun..

[25] Spranger S (2014). Mechanism of tumor rejection with doublets of CTLA-4, PD-1/PD-L1, or IDO blockade involves restored IL-2 production and proliferation of CD8(+) T cells directly within the tumor microenvironment. J. Immunother. Cancer.

[26] Long GV (2019). Epacadostat plus pembrolizumab versus placebo plus pembrolizumab in patients with unresectable or metastatic melanoma (ECHO-301/KEYNOTE-252): A phase 3, randomised, double-blind study. Lancet Oncol..

[27] Le Naour J, Galluzzi L, Zitvogel L, Kroemer G, Vacchelli E (2020). Trial watch: IDO inhibitors in cancer therapy. Oncoimmunology.

[28] Genard G, Lucas S, Michiels C (2017). Reprogramming of tumor-associated macrophages with anticancer therapies: Radiotherapy versus chemo- and immunotherapies. Front. Immunol..

[29] De Palma M, Lewis CE (2013). Macrophage regulation of tumor responses to anticancer therapies. Cancer Cell.

[30] Hoves S (2018). Rapid activation of tumor-associated macrophages boosts preexisting tumor immunity. J. Exp. Med..

[31] Gordon SR (2017). PD-1 expression by tumour-associated macrophages inhibits phagocytosis and tumour immunity. Nature.

[32] Chen W (2016). Attenuation of the programmed cell death-1 pathway increases the M1 polarization of macrophages induced by zymosan. Cell Death Dis.

[33] Dhupkar P, Gordon N, Stewart J, Kleinerman ES (2018). Anti-PD-1 therapy redirects macrophages from an M2 to an M1 phenotype inducing regression of OS lung metastases. Cancer Med..

[34] Cheng L, Wang Y, Huang L (2017). Exosomes from M1-polarized macrophages potentiate the cancer vaccine by creating a pro-inflammatory microenvironment in the lymph node. Mol. Ther..

[35] Pozzi LA, Maciaszek JW, Rock KL (2005). Both dendritic cells and macrophages can stimulate naive CD8 T cells in vivo to proliferate, develop effector function, and differentiate into memory cells. J. Immunol..

[36] Liu SH, Cheng YC (2012). Old formula, new Rx: The journey of PHY906 as cancer adjuvant therapy. J. Ethnopharmacol..

[37] Tan, H.Y. *et al.* Autophagy-induced RelB/p52 activation mediates tumour-associated macrophage repolarisation and suppression of hepatocellular carcinoma by natural compound baicalin. *Cell Death Dis.* **6**, 1942–1963 (2015). 10.1038/cddis.2015.271 PMC4632300 26492375

[38] Han X (2011). The development and functions of CD4(+) T cells expressing a transgenic TCR specific for an MHC-I-restricted tumor antigenic epitope. Cell Mol. Immunol..

[39] Weischenfeldt, J. & Porse, B. Bone marrow-derived macrophages (BMM): Isolation and applications. *CSH Protoc.* **2008**, 5080–5086 (2008). 10.1101/pdb.prot5080 21356739

[40] Braun D, Longman RS, Albert ML (2005). A two-step induction of indoleamine 2,3 dioxygenase (IDO) activity during dendritic-cell maturation. Blood.

