# Supplementary material for: YIV-906 potentiated anti-PD1 action against hepatocellular carcinoma by enhancing adaptive and innate immunity in the tumor microenvironment
Source: Sci Rep. 2021 Jun 29;11:13482. doi: 10.1038/s41598-021-91623-3 (PMC8242098; doi:10.1038/s41598-021-91623-3)
Supplement: Supplementary file 1 — Supplementary Information. [file 41598_2021_91623_MOESM1_ESM.pptx]

## Slide 1
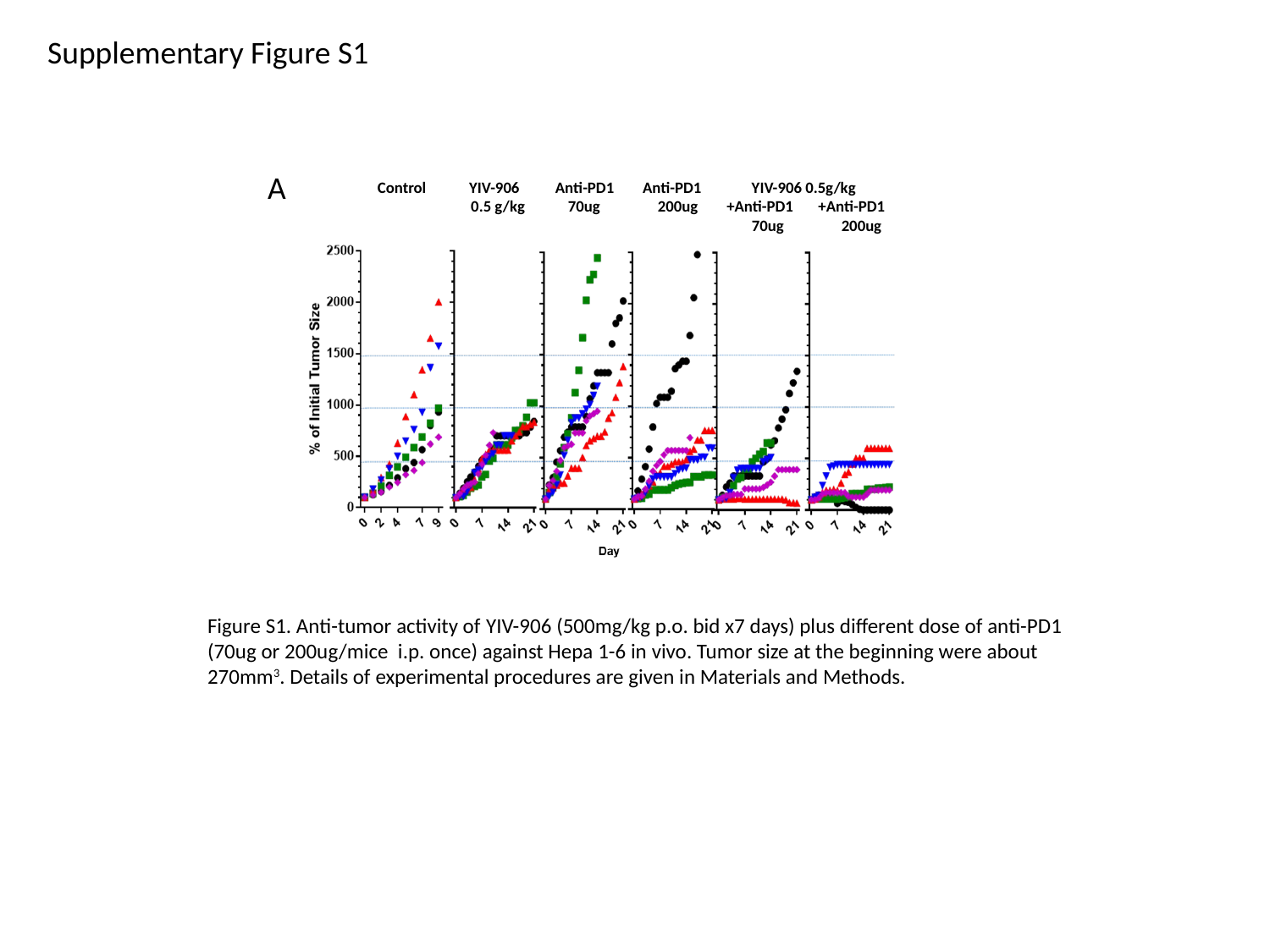

Supplementary Figure S1
A
Control YIV-906 Anti-PD1 Anti-PD1 YIV-906 0.5g/kg
 0.5 g/kg 70ug 200ug +Anti-PD1 +Anti-PD1
 70ug 200ug
Figure S1. Anti-tumor activity of YIV-906 (500mg/kg p.o. bid x7 days) plus different dose of anti-PD1 (70ug or 200ug/mice i.p. once) against Hepa 1-6 in vivo. Tumor size at the beginning were about 270mm3. Details of experimental procedures are given in Materials and Methods.

## Slide 2
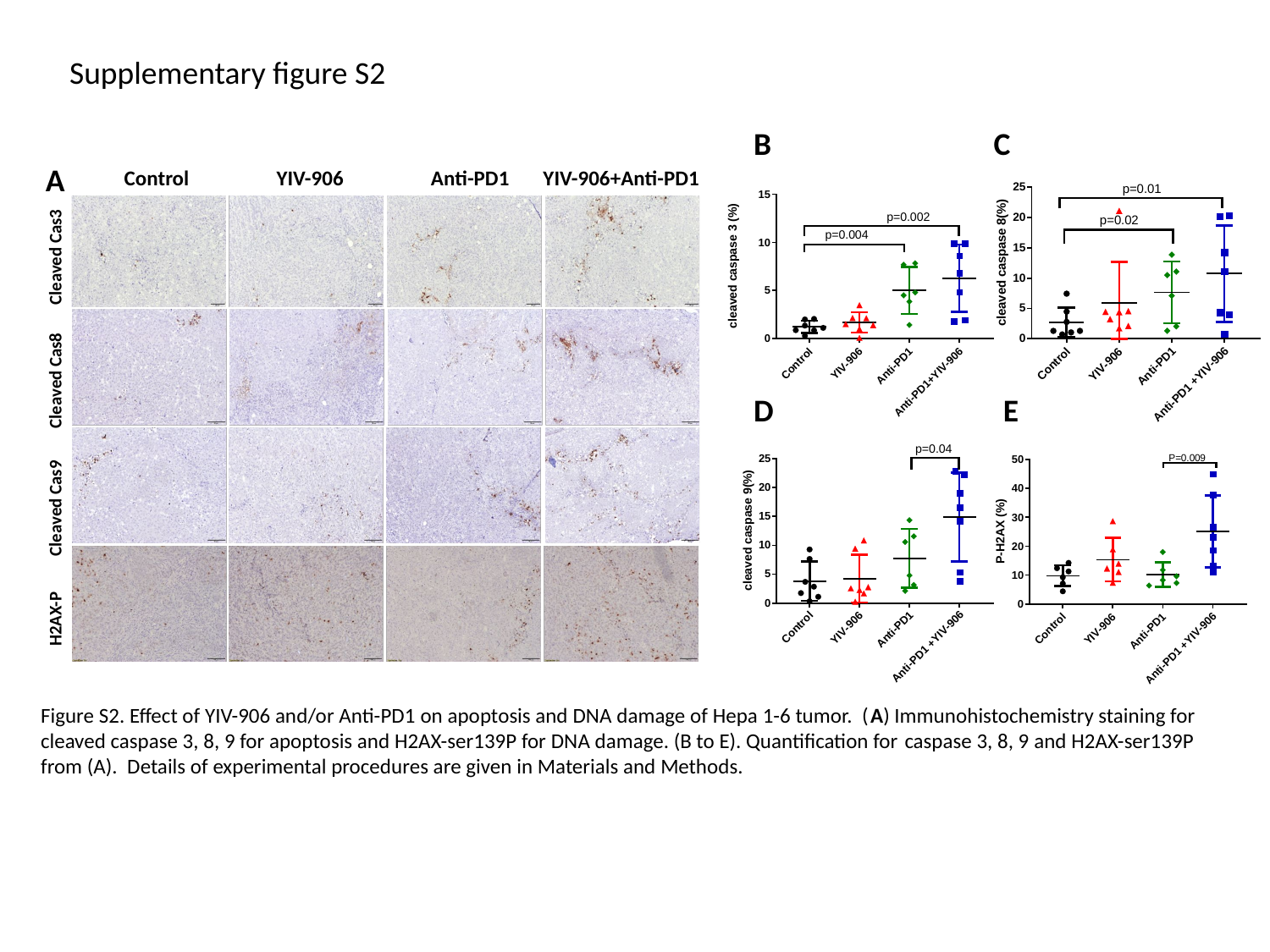

Supplementary figure S2
B C
D E
A
Control YIV-906 Anti-PD1 YIV-906+Anti-PD1
H2AX-P Cleaved Cas9 Cleaved Cas8 Cleaved Cas3
Figure S2. Effect of YIV-906 and/or Anti-PD1 on apoptosis and DNA damage of Hepa 1-6 tumor. (A) Immunohistochemistry staining for cleaved caspase 3, 8, 9 for apoptosis and H2AX-ser139P for DNA damage. (B to E). Quantification for caspase 3, 8, 9 and H2AX-ser139P from (A). Details of experimental procedures are given in Materials and Methods.

## Slide 3
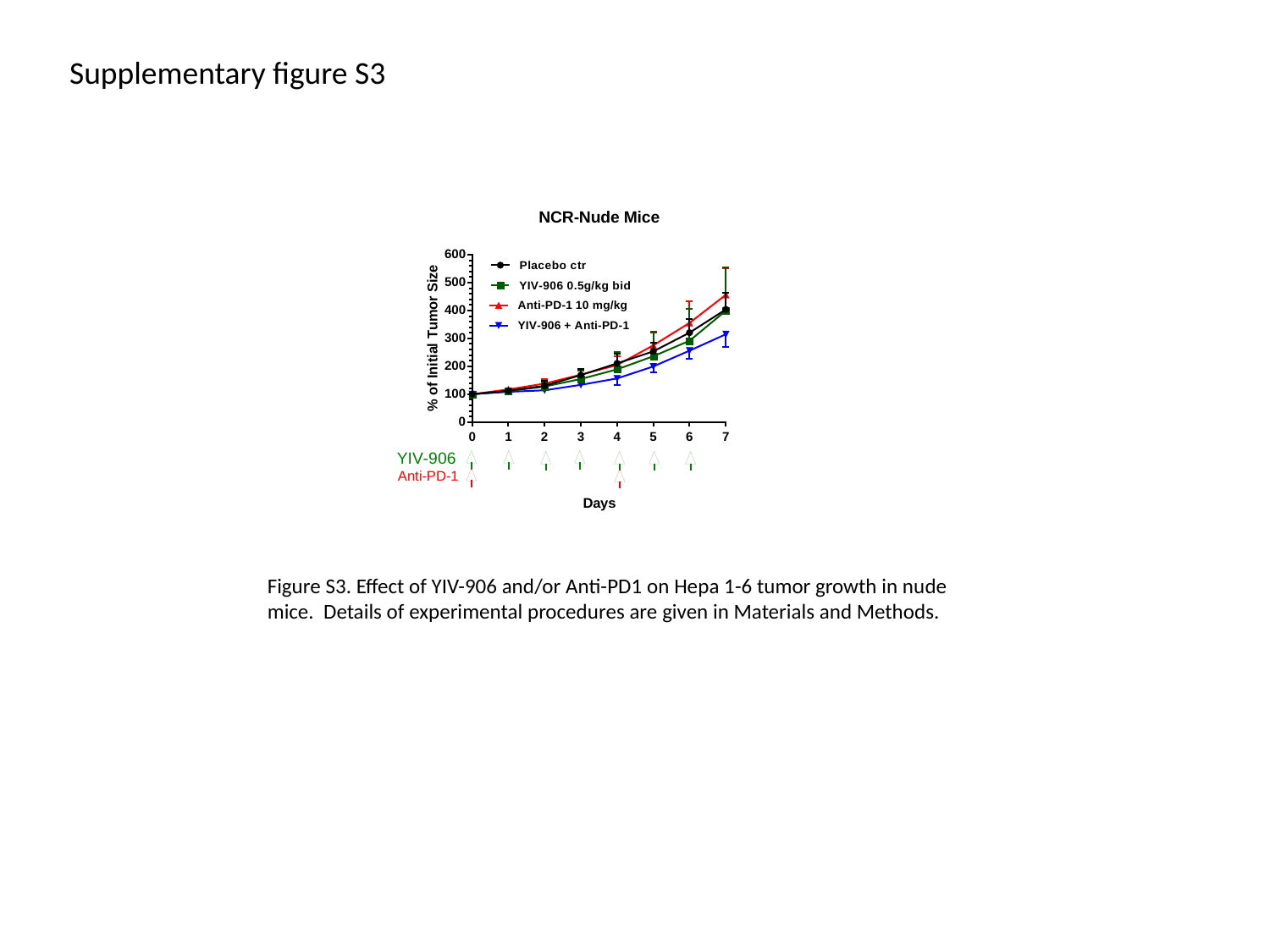

Supplementary figure S3
Figure S3. Effect of YIV-906 and/or Anti-PD1 on Hepa 1-6 tumor growth in nude mice. Details of experimental procedures are given in Materials and Methods.

## Slide 4
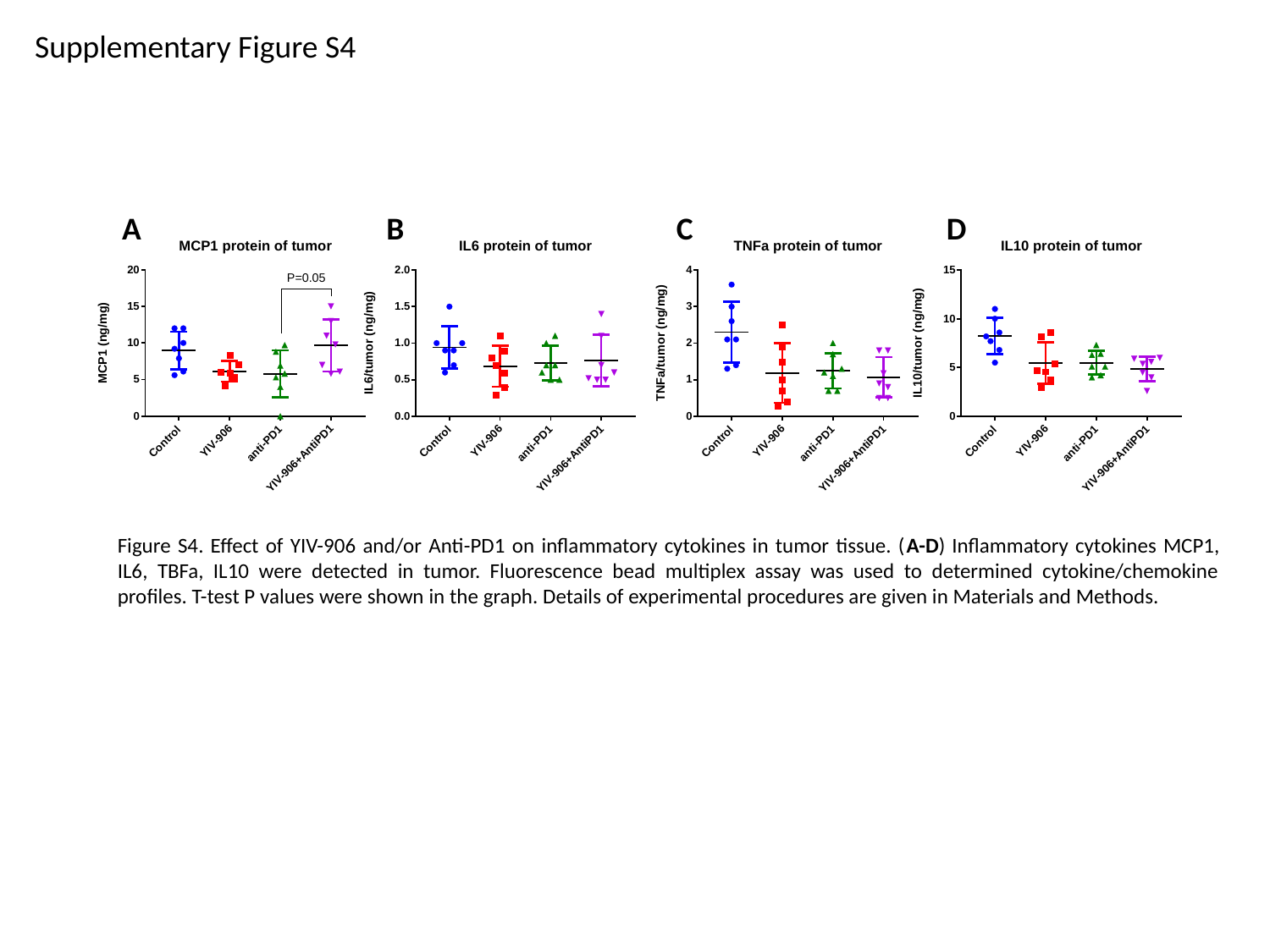

Supplementary Figure S4
A
B
C
D
Figure S4. Effect of YIV-906 and/or Anti-PD1 on inflammatory cytokines in tumor tissue. (A-D) Inflammatory cytokines MCP1, IL6, TBFa, IL10 were detected in tumor. Fluorescence bead multiplex assay was used to determined cytokine/chemokine profiles. T-test P values were shown in the graph. Details of experimental procedures are given in Materials and Methods.

## Slide 5
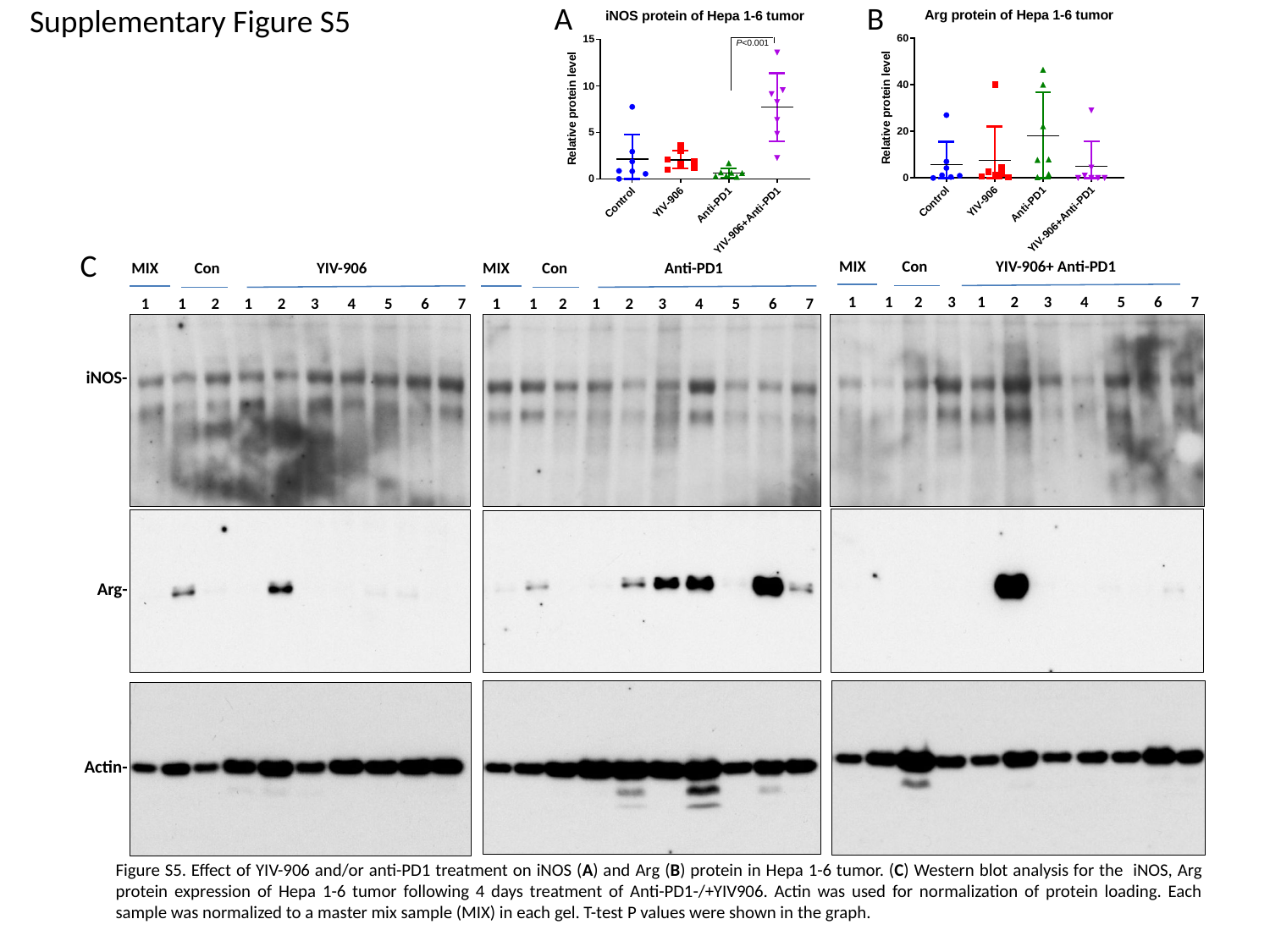

A B
Supplementary Figure S5
C
MIX Con YIV-906+ Anti-PD1
1 1 2 3 1 2 3 4 5 6 7
MIX Con YIV-906
1 1 2 1 2 3 4 5 6 7
MIX Con Anti-PD1
1 1 2 1 2 3 4 5 6 7
iNOS-
Arg-
Actin-
Figure S5. Effect of YIV-906 and/or anti-PD1 treatment on iNOS (A) and Arg (B) protein in Hepa 1-6 tumor. (C) Western blot analysis for the iNOS, Arg protein expression of Hepa 1-6 tumor following 4 days treatment of Anti-PD1-/+YIV906. Actin was used for normalization of protein loading. Each sample was normalized to a master mix sample (MIX) in each gel. T-test P values were shown in the graph.

## Slide 6
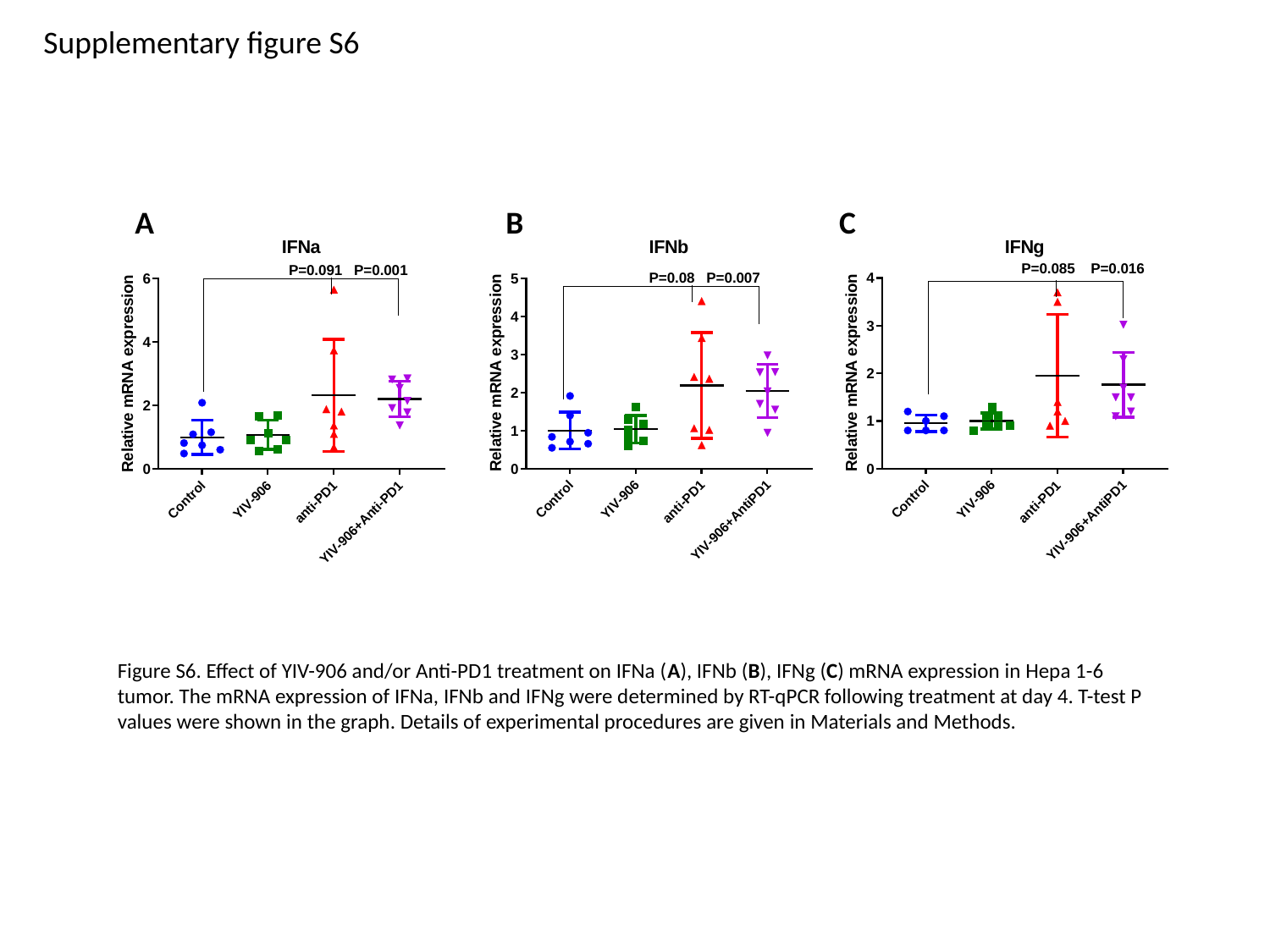

Supplementary figure S6
A B C
Figure S6. Effect of YIV-906 and/or Anti-PD1 treatment on IFNa (A), IFNb (B), IFNg (C) mRNA expression in Hepa 1-6 tumor. The mRNA expression of IFNa, IFNb and IFNg were determined by RT-qPCR following treatment at day 4. T-test P values were shown in the graph. Details of experimental procedures are given in Materials and Methods.

## Slide 7
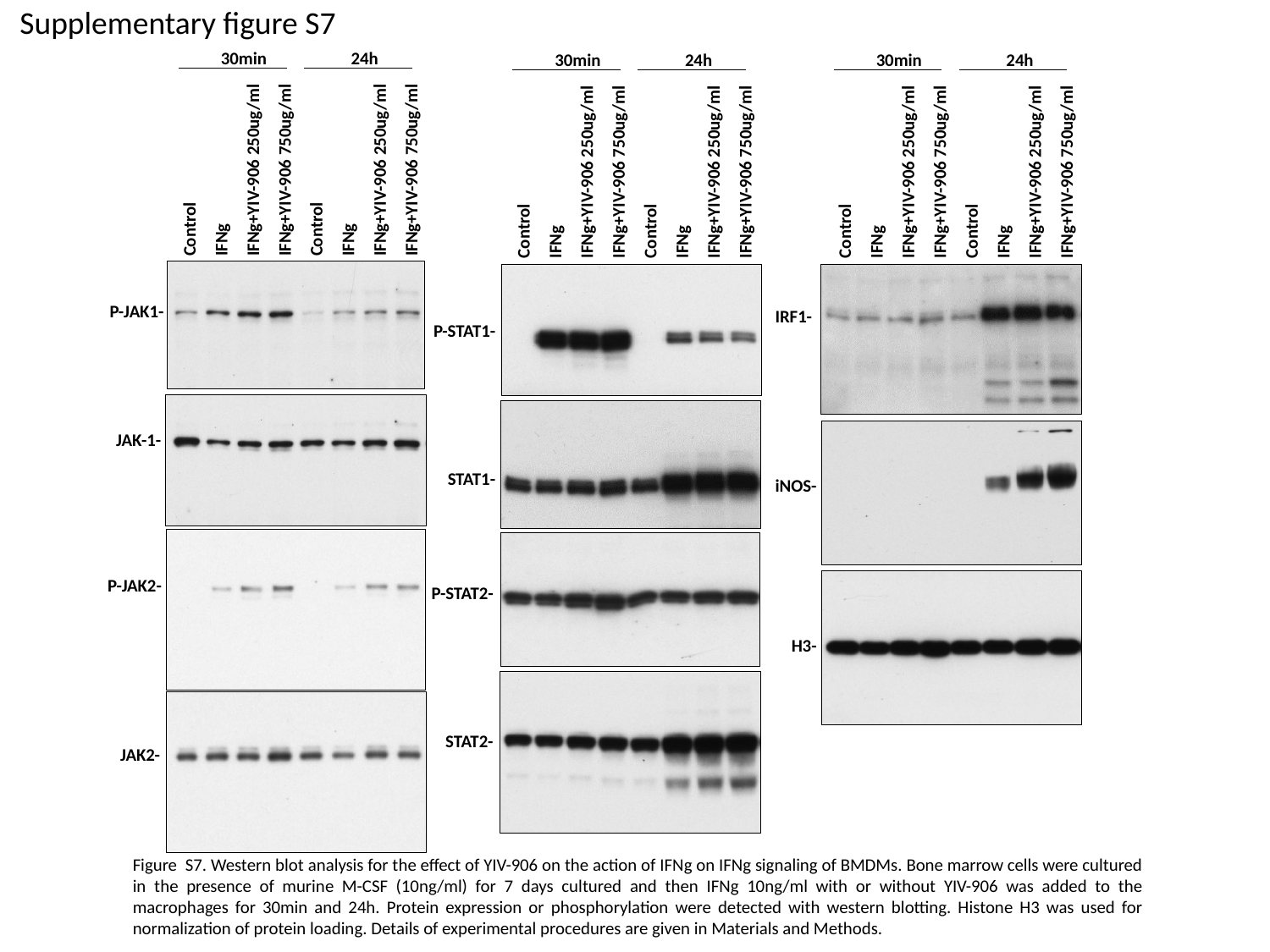

Supplementary figure S7
Control
IFNg
IFNg+YIV-906 250ug/ml
IFNg+YIV-906 750ug/ml
Control
IFNg
IFNg+YIV-906 250ug/ml
IFNg+YIV-906 750ug/ml
30min 24h
Control
IFNg
IFNg+YIV-906 250ug/ml
IFNg+YIV-906 750ug/ml
Control
IFNg
IFNg+YIV-906 250ug/ml
IFNg+YIV-906 750ug/ml
30min 24h
Control
IFNg
IFNg+YIV-906 250ug/ml
IFNg+YIV-906 750ug/ml
Control
IFNg
IFNg+YIV-906 250ug/ml
IFNg+YIV-906 750ug/ml
30min 24h
P-STAT1-
STAT1-
P-JAK1-
IRF1-
iNOS-
JAK-1-
P-JAK2-
P-STAT2-
STAT2-
H3-
JAK2-
Figure S7. Western blot analysis for the effect of YIV-906 on the action of IFNg on IFNg signaling of BMDMs. Bone marrow cells were cultured in the presence of murine M-CSF (10ng/ml) for 7 days cultured and then IFNg 10ng/ml with or without YIV-906 was added to the macrophages for 30min and 24h. Protein expression or phosphorylation were detected with western blotting. Histone H3 was used for normalization of protein loading. Details of experimental procedures are given in Materials and Methods.

## Slide 8
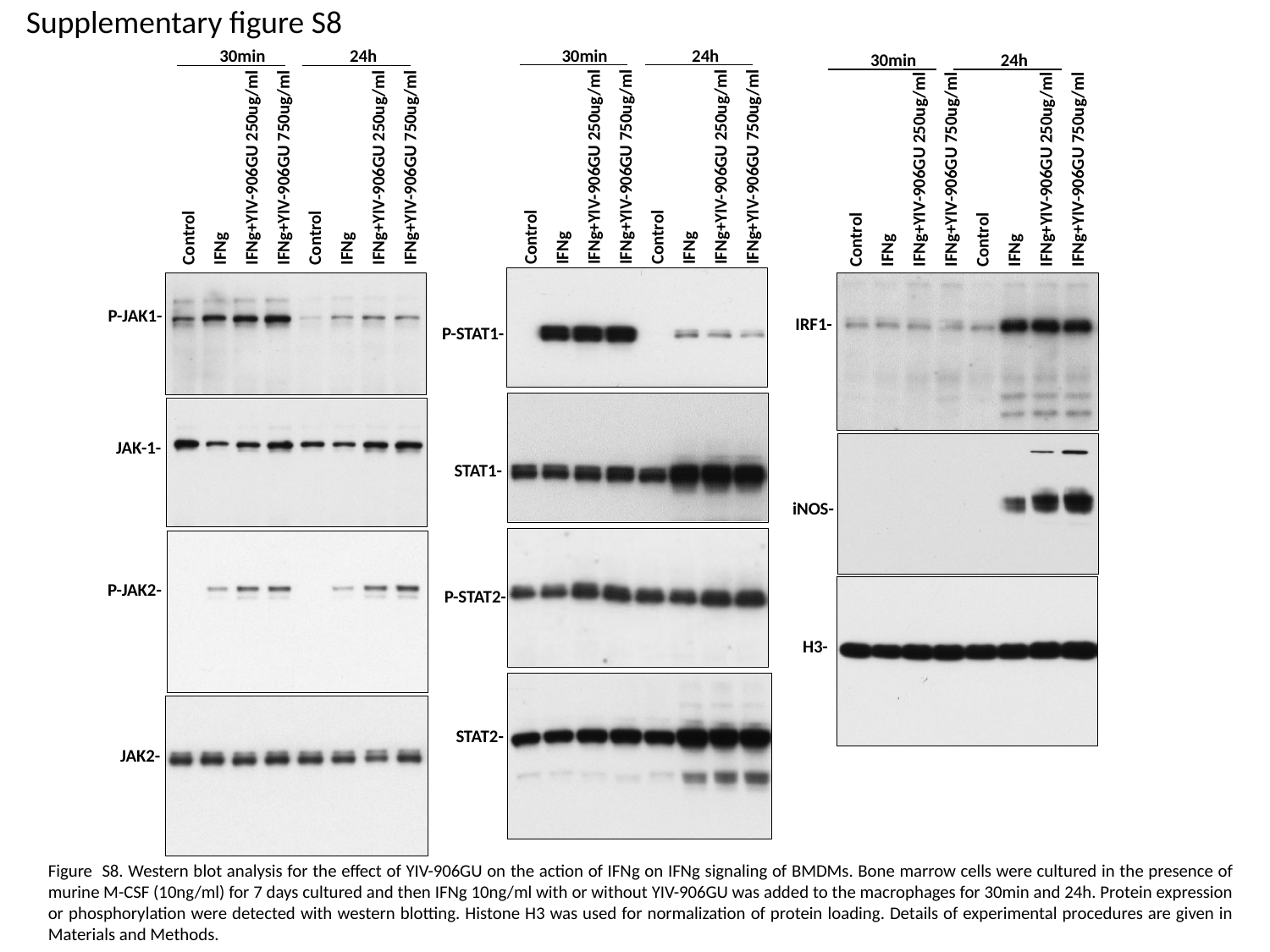

Supplementary figure S8
Control
IFNg
IFNg+YIV-906GU 250ug/ml
IFNg+YIV-906GU 750ug/ml
Control
IFNg
IFNg+YIV-906GU 250ug/ml
IFNg+YIV-906GU 750ug/ml
30min 24h
Control
IFNg
IFNg+YIV-906GU 250ug/ml
IFNg+YIV-906GU 750ug/ml
Control
IFNg
IFNg+YIV-906GU 250ug/ml
IFNg+YIV-906GU 750ug/ml
30min 24h
Control
IFNg
IFNg+YIV-906GU 250ug/ml
IFNg+YIV-906GU 750ug/ml
Control
IFNg
IFNg+YIV-906GU 250ug/ml
IFNg+YIV-906GU 750ug/ml
30min 24h
P-JAK1-
IRF1-
P-STAT1-
JAK-1-
STAT1-
iNOS-
P-JAK2-
P-STAT2-
H3-
STAT2-
JAK2-
Figure S8. Western blot analysis for the effect of YIV-906GU on the action of IFNg on IFNg signaling of BMDMs. Bone marrow cells were cultured in the presence of murine M-CSF (10ng/ml) for 7 days cultured and then IFNg 10ng/ml with or without YIV-906GU was added to the macrophages for 30min and 24h. Protein expression or phosphorylation were detected with western blotting. Histone H3 was used for normalization of protein loading. Details of experimental procedures are given in Materials and Methods.

## Slide 9
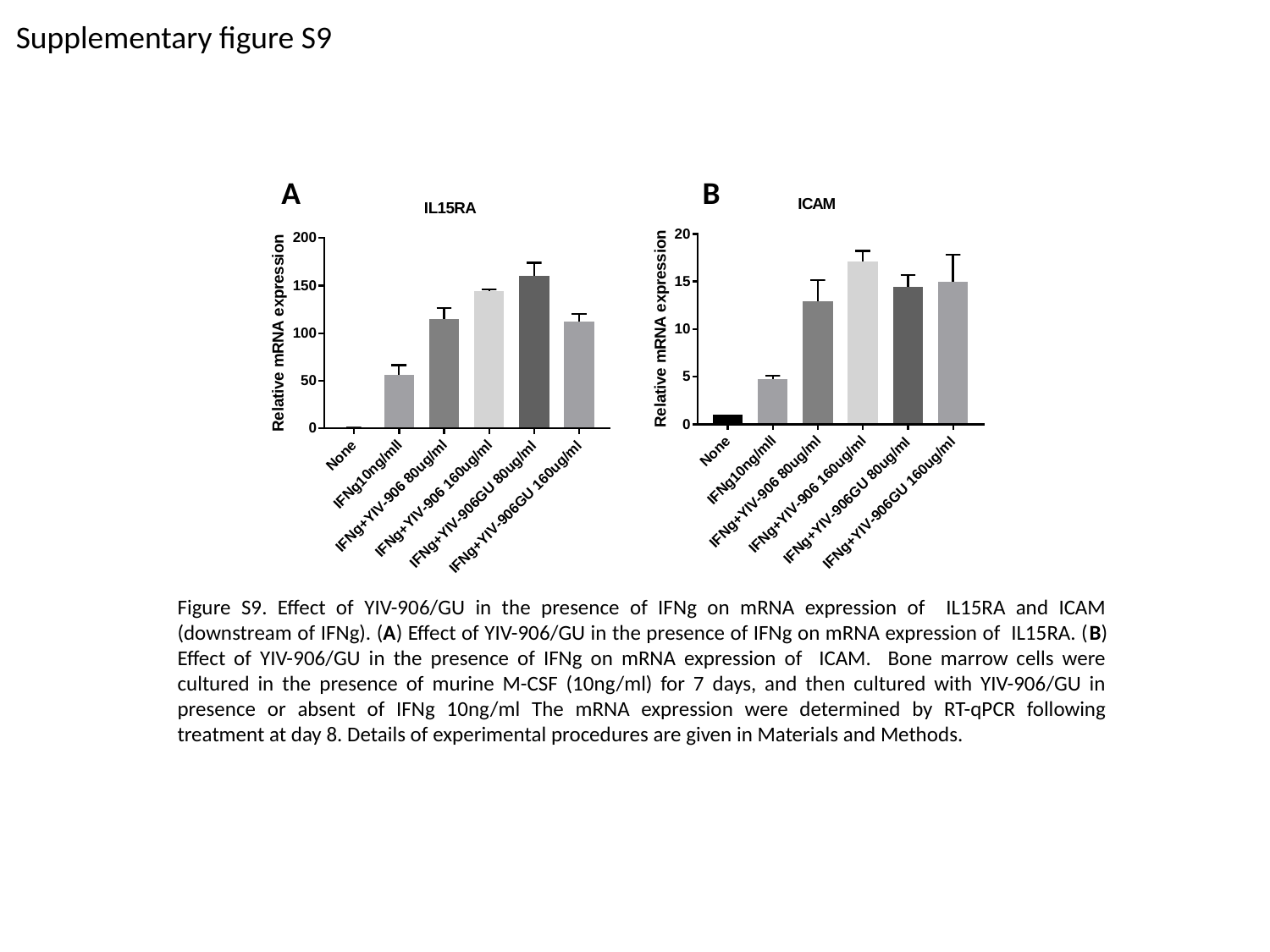

Supplementary figure S9
A B
Figure S9. Effect of YIV-906/GU in the presence of IFNg on mRNA expression of IL15RA and ICAM (downstream of IFNg). (A) Effect of YIV-906/GU in the presence of IFNg on mRNA expression of IL15RA. (B) Effect of YIV-906/GU in the presence of IFNg on mRNA expression of ICAM. Bone marrow cells were cultured in the presence of murine M-CSF (10ng/ml) for 7 days, and then cultured with YIV-906/GU in presence or absent of IFNg 10ng/ml The mRNA expression were determined by RT-qPCR following treatment at day 8. Details of experimental procedures are given in Materials and Methods.

## Slide 10
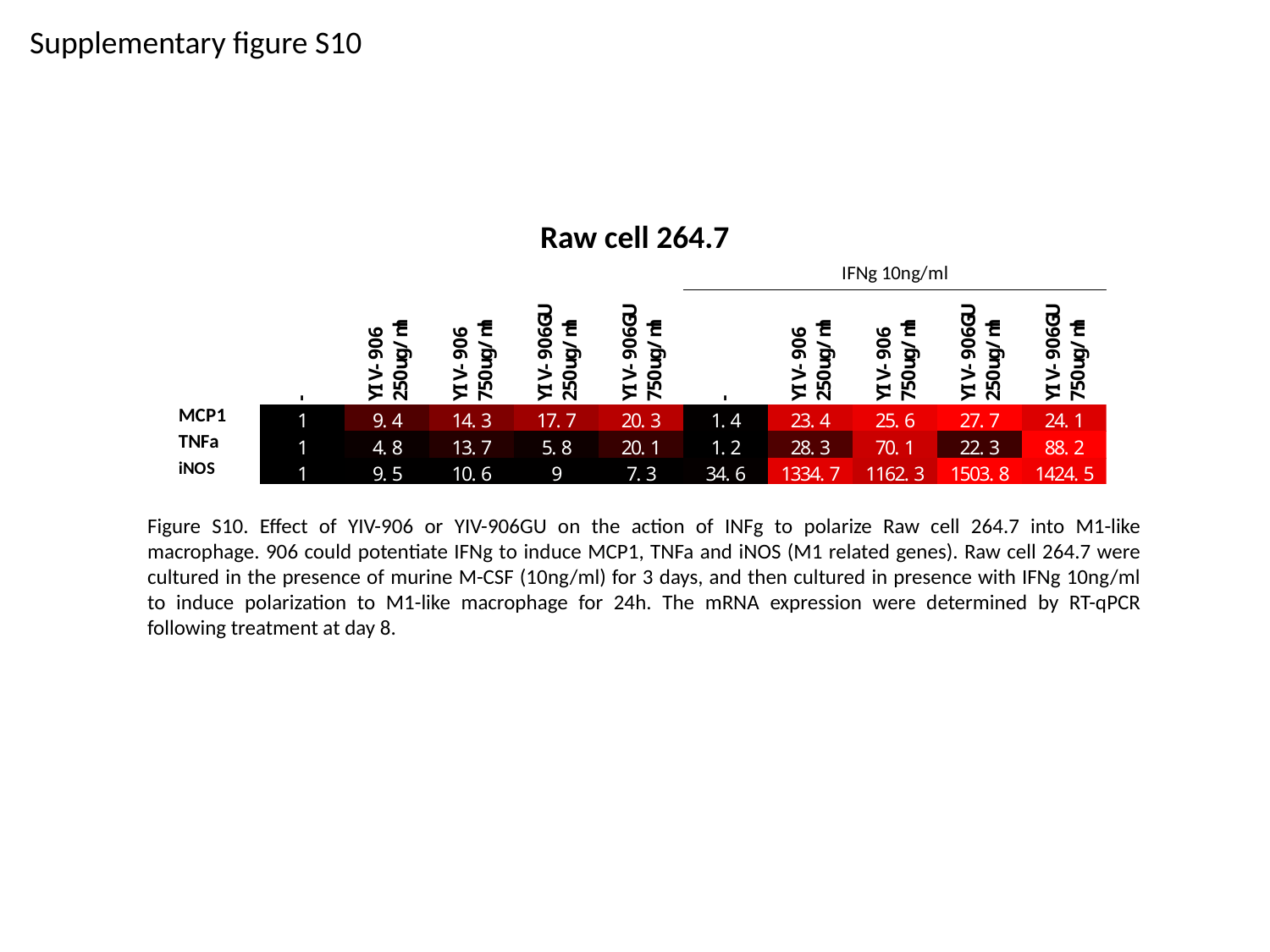

Supplementary figure S10
Raw cell 264.7
Figure S10. Effect of YIV-906 or YIV-906GU on the action of INFg to polarize Raw cell 264.7 into M1-like macrophage. 906 could potentiate IFNg to induce MCP1, TNFa and iNOS (M1 related genes). Raw cell 264.7 were cultured in the presence of murine M-CSF (10ng/ml) for 3 days, and then cultured in presence with IFNg 10ng/ml to induce polarization to M1-like macrophage for 24h. The mRNA expression were determined by RT-qPCR following treatment at day 8.

## Slide 11
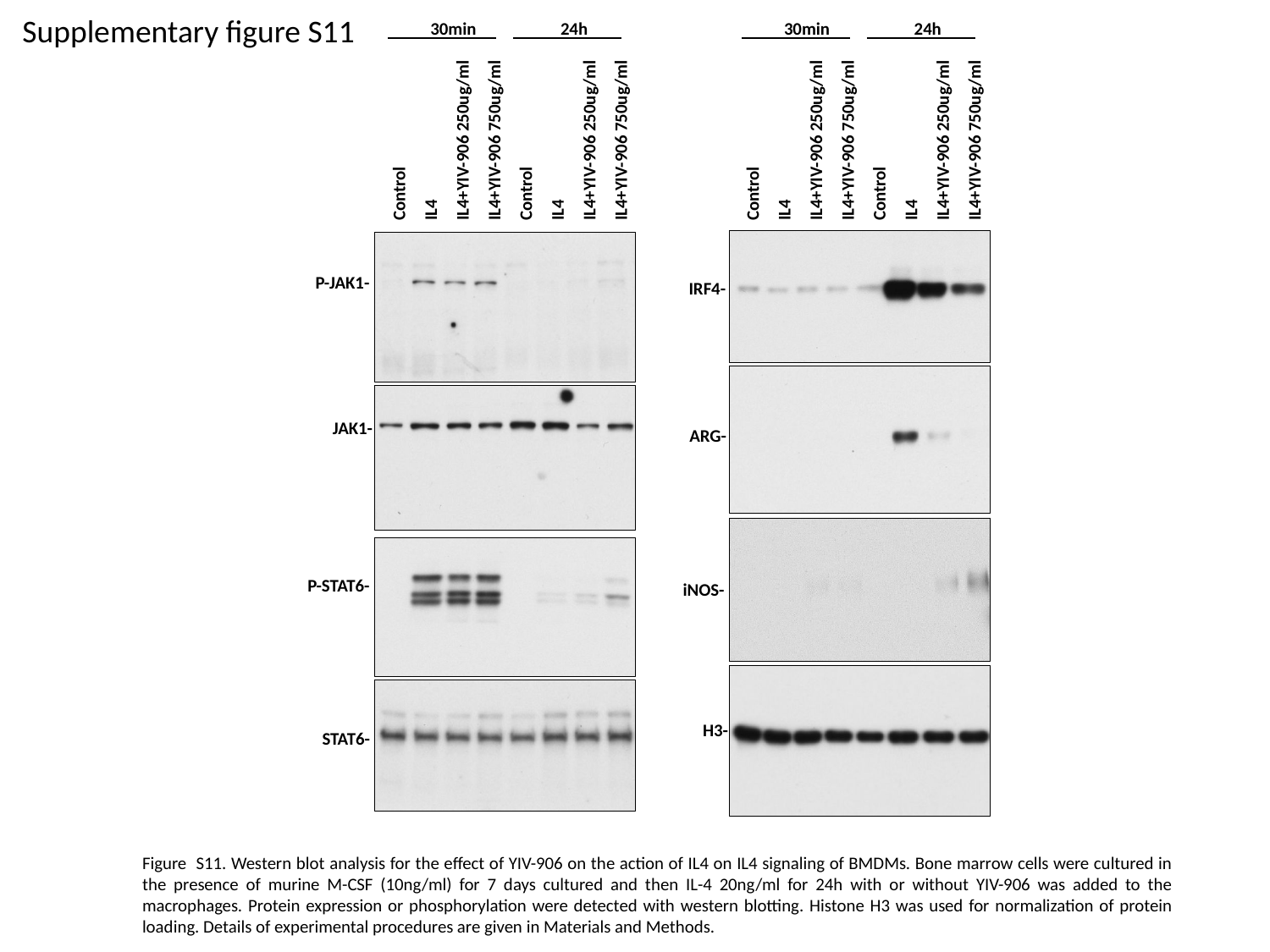

Supplementary figure S11
Control
IL4
IL4+YIV-906 250ug/ml
IL4+YIV-906 750ug/ml
Control
IL4
IL4+YIV-906 250ug/ml
IL4+YIV-906 750ug/ml
30min 24h
Control
IL4
IL4+YIV-906 250ug/ml
IL4+YIV-906 750ug/ml
Control
IL4
IL4+YIV-906 250ug/ml
IL4+YIV-906 750ug/ml
30min 24h
P-JAK1-
IRF4-
JAK1-
ARG-
P-STAT6-
iNOS-
H3-
STAT6-
Figure S11. Western blot analysis for the effect of YIV-906 on the action of IL4 on IL4 signaling of BMDMs. Bone marrow cells were cultured in the presence of murine M-CSF (10ng/ml) for 7 days cultured and then IL-4 20ng/ml for 24h with or without YIV-906 was added to the macrophages. Protein expression or phosphorylation were detected with western blotting. Histone H3 was used for normalization of protein loading. Details of experimental procedures are given in Materials and Methods.

## Slide 12
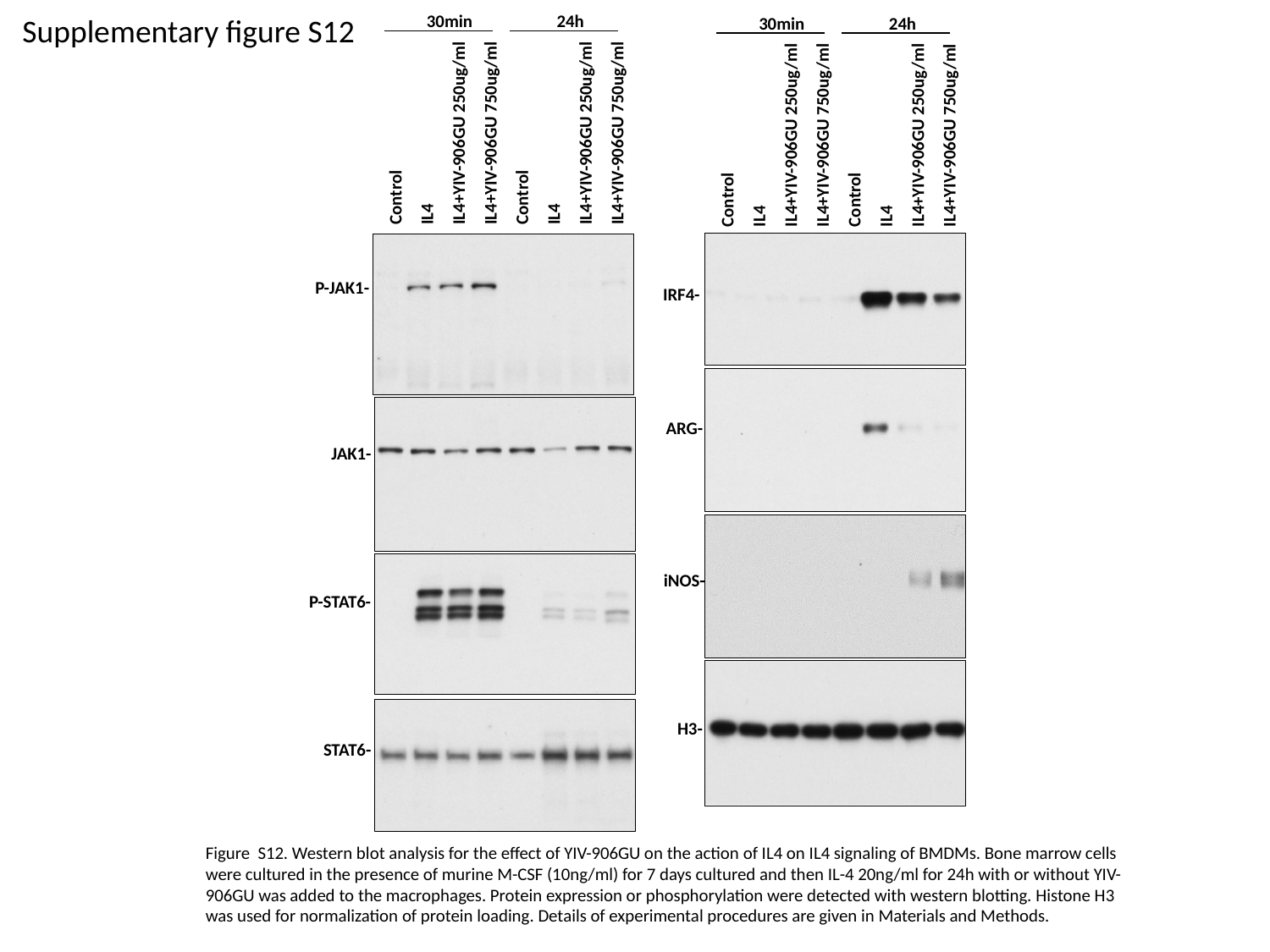

Control
IL4
IL4+YIV-906GU 250ug/ml
IL4+YIV-906GU 750ug/ml
Control
IL4
IL4+YIV-906GU 250ug/ml
IL4+YIV-906GU 750ug/ml
30min 24h
Supplementary figure S12
Control
IL4
IL4+YIV-906GU 250ug/ml
IL4+YIV-906GU 750ug/ml
Control
IL4
IL4+YIV-906GU 250ug/ml
IL4+YIV-906GU 750ug/ml
30min 24h
P-JAK1-
IRF4-
ARG-
JAK1-
iNOS-
P-STAT6-
H3-
STAT6-
Figure S12. Western blot analysis for the effect of YIV-906GU on the action of IL4 on IL4 signaling of BMDMs. Bone marrow cells were cultured in the presence of murine M-CSF (10ng/ml) for 7 days cultured and then IL-4 20ng/ml for 24h with or without YIV-906GU was added to the macrophages. Protein expression or phosphorylation were detected with western blotting. Histone H3 was used for normalization of protein loading. Details of experimental procedures are given in Materials and Methods.

## Slide 13
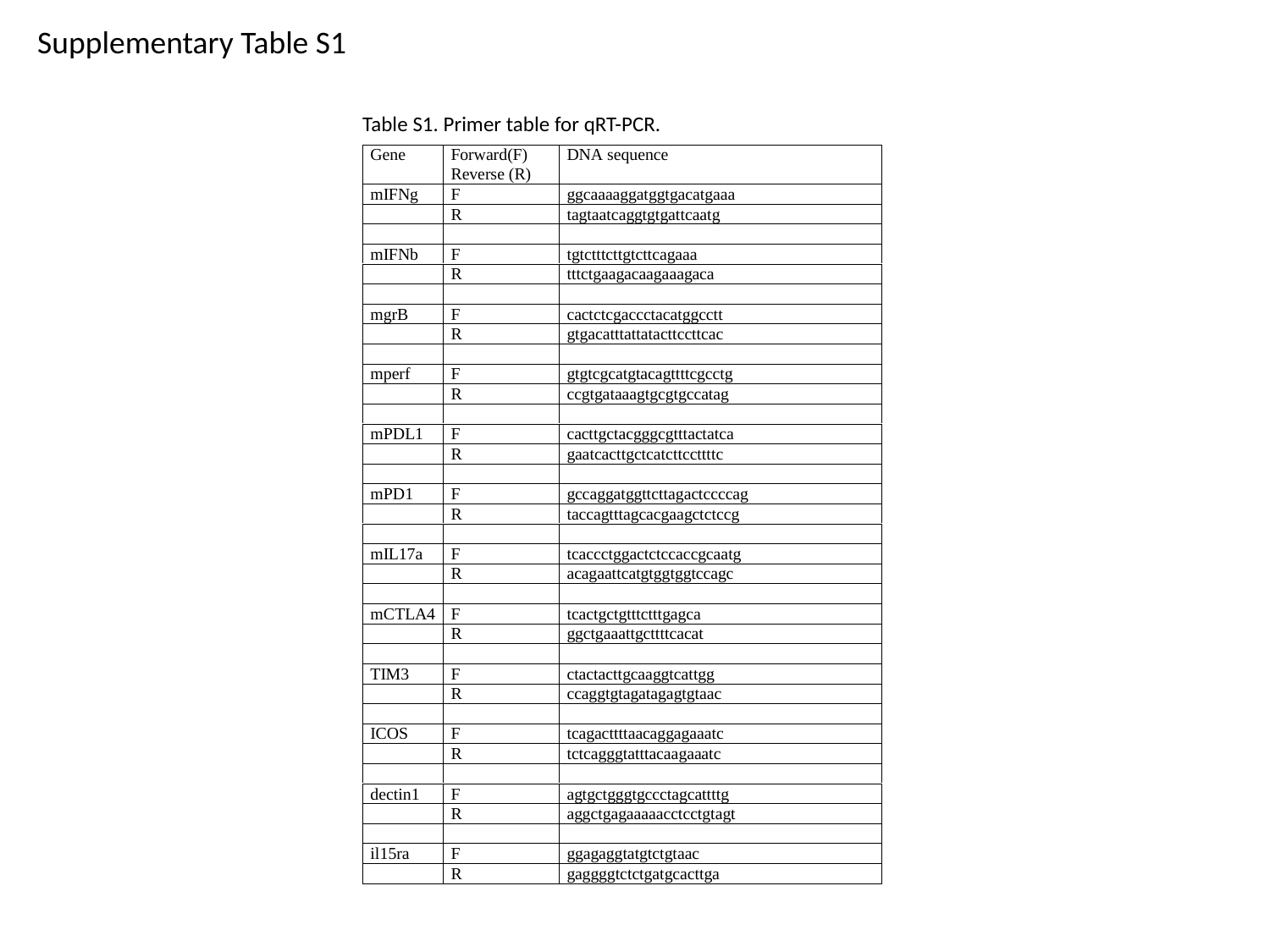

Supplementary Table S1
Table S1. Primer table for qRT-PCR.

## Slide 14
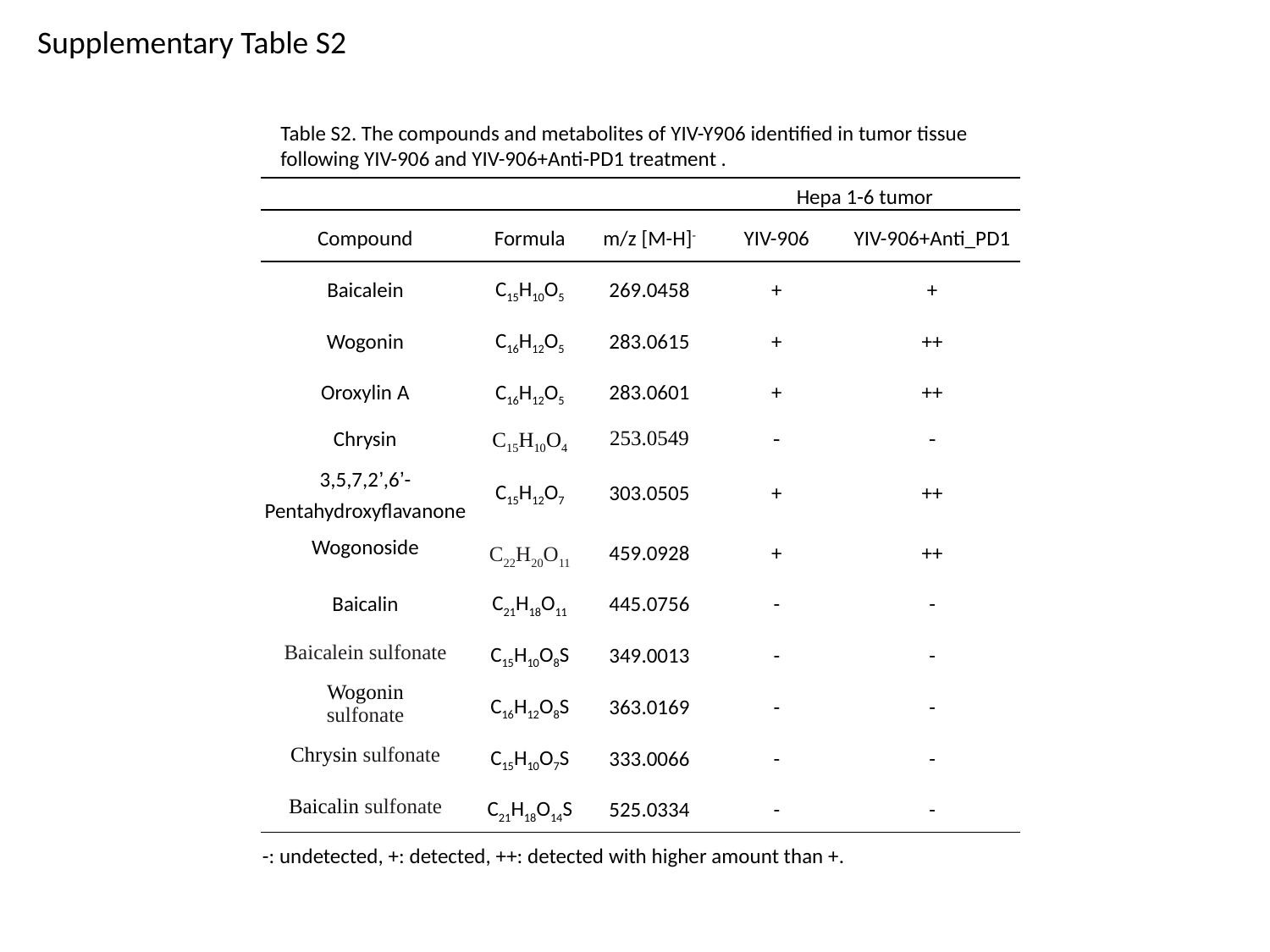

Supplementary Table S2
Table S2. The compounds and metabolites of YIV-Y906 identified in tumor tissue following YIV-906 and YIV-906+Anti-PD1 treatment .
| | | | Hepa 1-6 tumor | |
| --- | --- | --- | --- | --- |
| Compound | Formula | m/z [M-H]- | YIV-906 | YIV-906+Anti\_PD1 |
| Baicalein | C15H10O5 | 269.0458 | + | + |
| Wogonin | C16H12O5 | 283.0615 | + | ++ |
| Oroxylin A | C16H12O5 | 283.0601 | + | ++ |
| Chrysin | C15H10O4 | 253.0549 | - | - |
| 3,5,7,2’,6’-Pentahydroxyflavanone | C15H12O7 | 303.0505 | + | ++ |
| Wogonoside | C22H20O11 | 459.0928 | + | ++ |
| Baicalin | C21H18O11 | 445.0756 | - | - |
| Baicalein sulfonate | C15H10O8S | 349.0013 | - | - |
| Wogonin sulfonate | C16H12O8S | 363.0169 | - | - |
| Chrysin sulfonate | C15H10O7S | 333.0066 | - | - |
| Baicalin sulfonate | C21H18O14S | 525.0334 | - | - |
-: undetected, +: detected, ++: detected with higher amount than +.
